# Immunotherapy for Newly Diagnosed Glioblastoma: Current Evidence and Future Perspectives

**DOI:** 10.3390/jpm16090479

**Published:** 2026-09-17

**Authors:** Rudolfh Batista Arend, Bruno Zilli Peroni, Natan Lucca Lima, Miguel Cruz Garcia, Amanda Hedel Koerich, André Felipe do Nascimento, Rafael Torres Fonseca dos Santos, Kelvin Tapia, Daniel Kerpel, Gustavo Simiano Jung, Alex Roman, Guilherme Gago, Martin Batista Coutinho da Silva, Antonio Delacy Martini Vial, Edoardo Agosti

**Affiliations:** 1 Faculty of Medicine, Federal University of Fronteira Sul, Passo Fundo 99010-200, RS, Brazil; rudolfharend77@gmail.com (R.B.A.); bruno.peroni@hotmail.com (B.Z.P.); alexroman__@hotmail.com (A.R.); 2 Faculty of Medicine, Federal University of Santa Catarina, Araranguá 99010-200, SC, Brazil; 3 Faculty of Medicine, Institute of Medical Education, Rio de Janeiro 20071-004, RJ, Brazil; miguelcgarciamd@gmail.com (M.C.G.);; 4 Faculty of Medicine, University of Passo Fundo, Passo Fundo 99052-900, RS, Brazil; 5 Faculty of Medicine, Universidad Adventista del Plata (UAP), Libertador San Martín E3103XAF, Entre Ríos, Argentina; kelvinbtborges@gmail.com; 6 Faculty of Medicine, Federal University of Santa Maria, Santa Maria 97105-900, RS, Brazil; 7 Department of Neurosurgery, Neurological Institute of Curitiba, Curitiba 82821-020, PR, Brazil; 8 Department of Neurosurgery, Université Laval, Quebec City, QC G1V 0A6, Canada; guilherme.gago@gmail.com; 9 Department of Medicine, University of Passo Fundo, Passo Fundo 99052-900, RS, Brazil; martinbcoutinho@gmail.com; 10 Department of Neurosurgery, Irmandade Santa Casa de Misericórdia de Porto Alegre, Hospital São José, Porto Alegre 90040-020, RS, Brazil; 11 Department of Medical and Surgical Specialties, Division of Neurosurgery, Radiological Sciences and Public Health, University of Brescia, Lombardy, 25121 Brescia, Italy

**Keywords:** dendritic cell vaccines, glioblastoma, immune checkpoint inhibitors, peptide vaccines, target therapies

## Abstract

**Background**: Glioblastoma (GBM) represents the most aggressive and lethal primary malignancy of the central nervous system in adults. Although clinical trials evaluating immunotherapy strategies have increased substantially in recent years, the available evidence remains highly fragmented. This scoping review aimed to synthesize the available evidence from clinical trials investigating immunotherapy for newly diagnosed GBM. **Methods**: PubMed, Scopus, Embase, and Web of Science were searched from inception to July 2026 for clinical trials of first-line immunotherapy in adults with newly diagnosed GBM; recurrent, mixed-setting, preclinical, and abstract-only reports were excluded. Outcomes were overall survival (OS) and progression-free survival (PFS), adverse events, and treatment-related deaths. **Results**: Six therapeutic classes were represented: immune checkpoint inhibitors (13 reports), peptide and neoantigen vaccines (12), dendritic cell vaccines (10), adoptive cell therapies (5), oncolytic viral and gene therapies (3), and autologous formalin-fixed tumor vaccines (2). The evidence base was predominantly early-phase: 36 of 45 studies enrolled fewer than 100 patients, only 14 included a randomized comparison, and 5 were phase III. Median OS ranged from 11.5 to 38.4 months and median PFS from 1.3 to 60.1 months, the highest estimates arising from small, highly selected single-arm cohorts. No randomized trial improved OS. Important adverse events were rare and almost exclusively confined to checkpoint inhibitor trials. **Conclusions**: Immunotherapy in newly diagnosed GBM is feasible and tolerable but has not improved survival, with outcomes still determined more by MGMT promoter status and extent of resection than by treatment assignment. Biomarker-enriched selection, lymphocyte-sparing design and rational combinations are required in future trials.

## 1. Introduction

Glioblastoma (GBM), an IDH-wildtype CNS WHO grade 4 tumor, represents the most aggressive and lethal primary malignancy of the central nervous system in adults. Despite advances in neuro-oncology, GBM remains associated with a dismal prognosis, with a median overall survival (OS) of less than one year from diagnosis. The current standard of care for newly diagnosed GBM consists of maximal safe surgical resection followed by concurrent radiotherapy and temozolomide chemotherapy, with subsequent adjuvant temozolomide [1]. However, disease recurrence is nearly universal and is associated with poor clinical outcomes, with reported median OS ranging from 2 to 9 months and progression-free survival (PFS) from 1.5 to 6 months following recurrence [2]. As first-line treatment has remained largely unchanged for nearly two decades and continues to provide only modest survival benefits, there is a pressing need to develop and integrate novel therapeutic strategies into the initial management of newly diagnosed GBM.

In this context, immunotherapy has transformed the treatment of several solid malignancies and is now being actively investigated as a potential therapeutic strategy for GBM. Approaches including immune checkpoint inhibitors, therapeutic vaccines, adoptive cell therapies, and oncolytic viruses have all entered clinical evaluation [3]. However, the clinical benefit observed in other cancers has not yet been reproduced in GBM, largely because of the tumor’s unique biology. The blood–brain barrier, marked intratumoral heterogeneity, low immunogenicity, and a profoundly immunosuppressive tumor microenvironment all limit the effectiveness of antitumor immune responses [3]. While most immunotherapy trials have historically focused on recurrent disease, attention has recently shifted toward newly diagnosed GBM, where treatment may be initiated before extensive therapy-induced immune dysfunction develops [4].

Although clinical trials evaluating these early interventions have increased substantially in recent years, the available evidence remains highly fragmented [5]. Studies differ significantly in the immunotherapeutic strategies investigated, trial design, treatment sequencing, and integration with standard-of-care therapy. This heterogeneity makes it difficult to obtain a comprehensive understanding of the field and to identify the most promising therapeutic approaches. Therefore, this scoping review aims to characterize and synthesize the available evidence from human clinical trials investigating immunotherapy for newly diagnosed GBM. We also aim to describe current therapeutic strategies, examine their integration with gold-standard treatments, and identify methodological gaps to inform the design of future clinical research.

## 2. Materials and Methods

This scoping review was registered on Figshare under protocol https://doi.org/10.6084/m9.figshare.33189945, and the final methodology was consistent with the pre-registered protocol, with no major deviations. A scoping review was chosen and registered as such because the body of evidence is heterogeneous in intervention, trial design, comparator, outcome definition, and response criteria to a degree that precludes meaningful pooling; the purpose is to map what has been done and what has not, rather than to estimate a treatment effect. The review was conducted following the Joanna Briggs Institute (JBI) Manual for Evidence Synthesis and reported according to the PRISMA Extension for Scoping Reviews (PRISMA-ScR) [6,7].

### 2.1. Defining the Research Question

The overarching research question guiding this review was: ‘What is the current landscape of clinical trials investigating immunotherapeutic strategies for newly diagnosed glioblastoma?’ To address this question comprehensively, the review investigated four specific sub-questions:

1. Which immunotherapeutic modalities have been evaluated in clinical trials for newly diagnosed glioblastoma?
2. What are their reported mechanisms of action, clinical efficacy, and safety profiles?
3. Which predictive or prognostic biomarkers have been assessed within these trial cohorts?
4. What primary clinical endpoints are reported, and what major research gaps persist in the current literature?

### 2.2. Eligibility Criteria

Eligibility criteria were developed according to the Population, Concept, and Context (PCC) framework recommended by JBI. The population of interest included adult patients with newly diagnosed GBM confirmed by histopathological or molecular criteria. The concept of interest was the use of immunotherapeutic interventions, including immune checkpoint inhibitors, therapeutic vaccines, adoptive cell therapies, oncolytic viruses, and other immune-based strategies administered as part of first-line treatment. The context was limited to human clinical trials evaluating immunotherapy in the newly diagnosed setting, either as monotherapy or in combination with standard of care.

A study was included if it involved adults with newly diagnosed GBM or CNS WHO grade 4 astrocytoma confirmed histopathologically or molecularly, who were not treated before. The study had to administer an immunotherapeutic intervention, which could include immune checkpoint inhibitors; various vaccines such as peptide, dendritic cell, neoantigen, DNA, RNA, tumor lysate, or whole tumor cell vaccines; adoptive cell therapies including CAR-T, CAR-NK, TCR-T, tumor-infiltrating lymphocytes, cytokine-induced killer, and lymphokine-activated killer cells; oncolytic viruses; therapeutic cytokines; bispecific antibodies and T-cell engagers; immunotoxins; TLR or STING agonists; or immune gene therapy, either alone or in combination with standard of care. Eligible studies were human clinical trials of any phase, randomized or single-arm, without restrictions by country or year. For mixed cohorts, at least 75% of participants had to have newly diagnosed GBM or the subgroup had to be reported separately.

Studies were excluded if the cohort consisted of >25% patients with recurrent, progressive, or refractory disease; if the cohort was exclusively pediatric; or if the cohort involved diffuse intrinsic pontine glioma, or tumors other than GBM or CNS WHO grade 4 astrocytoma, with anaplastic astrocytoma and grade 3 glioma not accepted as equivalents. Studies were also excluded if immunotherapy was only proposed or discussed but not administered, if they were preclinical works such as animal, in vitro, cell line, or in silico studies, or if they were narrative reviews, systematic reviews, meta-analyses, editorials, commentaries, letters without original data, or guidelines. Protocols and trial registry entries without results, conference abstracts, case reports, and case series with fewer than five patients conducted outside protocolled trials were excluded, as were duplicate publications that added no new data to included primary reports.

### 2.3. Search Strategy

The databases (PubMed, Scopus, Embase, Web of Science, and ClinicalTrials.gov databases) were searched from inception to July 2026 in an independent manner by two reviewers (M.C.G. and R.B.A.). The following search strategy was used: (glioblastoma OR \”glioblastoma multiforme\” OR GBM) AND (immunotherap\ OR \”immune checkpoint inhibitor\\” OR \”checkpoint inhibitor\\” OR vaccin\ OR \”dendritic cell\\” OR \”CAR T\” OR CAR-T OR \”adoptive cell therap\\” OR \”natural killer cell\\” OR NK OR \”oncolytic virus\\” OR virother\ OR cytokine\).

### 2.4. Selection of Sources of Evidence

The selection of sources of evidence was conducted through a two-stage screening process (title/abstract and full text). This process was conducted independently by two reviewers (M.C.G. and R.B.A.) using Rayyan Software (Rayyan Systems, Cambridge, MA, USA; web-based platform). In case of any disagreements, a third reviewer (B.Z.P.) was consulted to make the final decision.

### 2.5. Data Charting

Data were charted by two authors (A.H.K. and A.F.N.) following predefined eligibility criteria. These extracted data included study characteristics (first author, year of publication, country, study design, multi/single center, findings, methodology, and objectives), population characteristics (eligibility criteria, total number of patients, mean or median age, sex distribution, etc.), tumor characteristics (new or recurrent GBM, extent of resection, IDH status, other biomarkers, concomitant therapies), therapy (agent, molecular target, posology, etc.), and oncological outcomes (OS, PFS, adverse effects, discontinuation, etc). Additionally, a synthesis of the results was elaborated for each study.

### 2.6. Analysis and Presentation of Results

The extracted data were mapped and presented using a combination of tabular and diagrammatic formats, complemented by a structured narrative summary designed to directly address the review questions. Visual aids illustrate the overarching characteristics of the included evidence, detailing the distribution of sources by publication year, country of origin, intervention context, and research methodology. To facilitate a robust synthesis, the findings were organized into core conceptual categories: population characteristics (including sample sizes), intervention types and durations, study objectives, methodological approaches, primary findings (established evidence), and identified research gaps. A comprehensive narrative explanation accompanies each of these domains, explicitly articulating how the charted data align with the overarching objective and foundational questions of this scoping review.

## 3. Results

### 3.1. Study Selection

**Figure 1:**
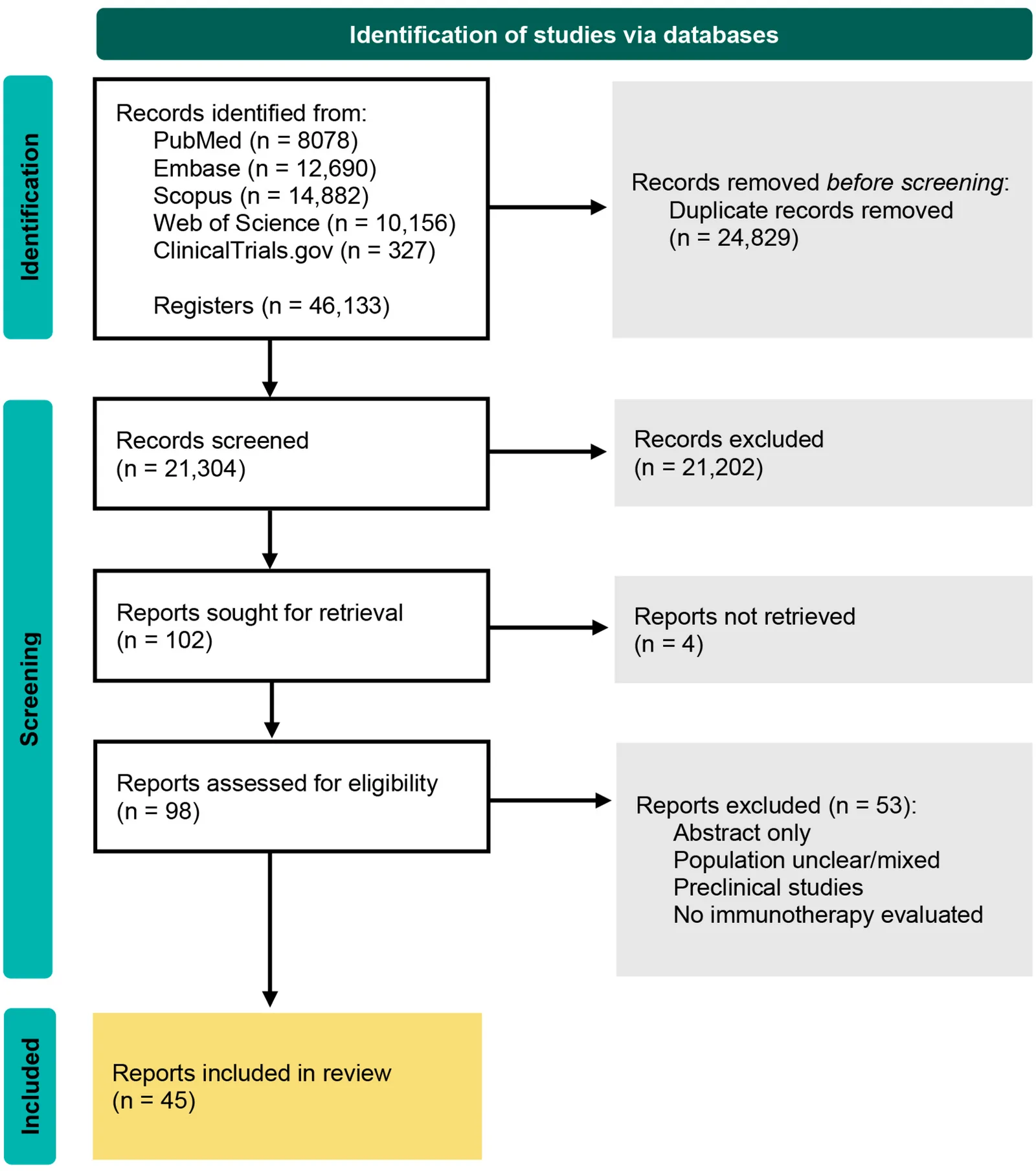
PRISMA flow diagram.

The database search retrieved 21,304 records, which were screened at title and abstract level. Ninety-eight reports were retained for full-text assessment, of which 45 met the eligibility criteria and were included in the review. The selection process is illustrated in the PRISMA flow diagram (Figure 1).

### 3.2. Baseline Characteristics of Included Studies

Forty-five reports of immunotherapy in newly diagnosed GBM met the inclusion criteria and are summarized by modality in Table 1 and study by study in Table S1. These 45 reports described 41 distinct clinical trials. Four were companion publications of a trial already represented: two correlative analyses of the heat shock protein peptide complex-96 trial (NCT02122822), examining T-cell receptor repertoire and MxA expression against outcome [8,9]; a post hoc subgroup analysis of the cytokine-induced killer trial (NCT00807027) restricted to pathologically pure GBM [10]; and a second record charted from the nivolumab plus IDO1 inhibitor trial (NCT04047706) [11]. The two reports by Ardon et al. were treated as separate trials. The 2010 report details an eight-patient pilot whose six-month progression-free survival rate powered the HGG-2006 trial [12], which enrolled 77 patients from 2006 to 2008 [13]. Since a trial cannot be powered by its own participants, the HGG-2006 report clearly separates the pilot from the main trial, contrasting their immune monitoring. Both used the same vaccine and radiochemoimmunotherapy schedule, with later vaccines prepared as previously described. Thus, the pilot was a feasibility study, not a subgroup of the main trial. Four additional reports [14,15,16,17] are secondary analyses of trials excluded from the review. Companion sets are noted in Table S1, and the synthesis unit is the trial, not the article.

The evidence base is dominated by early-phase, single-center experience: seventeen reports described phase I trials and six phase I/II trials, whereas only five studies were phase III, while one was a seamless phase II/III design. Eighteen studies were conducted at a single center and twenty-seven were multicenter, with the number of participating sites ranging from 2 to 165. Most trials were run in North America (22 studies conducted exclusively in the USA), followed by Europe and East Asia; six studies recruited internationally across as many as 22 countries. Sample sizes varied significantly, ranging between 4 and 745 participants. Thirty-six of the forty-five reports (80.0%) enrolled fewer than 100 participants, and twenty-four (53.3%) enrolled fewer than 35. Only 14 studies included a randomized comparison, and blinding was uncommon. Participants were newly diagnosed GBM patients. Median age clustered between 50 and 61 years across most studies. Women were consistently under-represented, accounting for approximately 35% of participants, consistent with the known male predominance of GBM. Detailed data can be found in Table S1.

Table 2 summarizes the disease status and immunotherapeutic intervention of each study. The criteria governing eligibility for the review itself are set out in Section 2.2. Eligibility within the included trials was highly selective and remarkably consistent: nearly all protocols required histologically confirmed, newly diagnosed GBM after maximal safe resection; a Karnofsky performance status of at least 60; adequate hematological, hepatic, and renal function; and low or tapering corticosteroid doses. Additional restrictions were frequently imposed by the immunotherapeutic platform itself. Populations were almost exclusively composed of newly diagnosed disease: 42 of the 45 reports enrolled 100% newly diagnosed GBM, and no study included recurrent disease as its primary population. The three exceptions comprised mixed high-grade glioma cohorts in which GBM accounted for 80% [18], 90% [19], and 92% [20] of participants.

Six therapeutic classes were represented. Immune checkpoint inhibitors, alone or in combination, were evaluated in thirteen reports and targeted PD-1, PD-L1, CTLA-4, or IDO1; peptide and neoantigen vaccines accounted for twelve reports and targeted EGFRvIII, survivin, IDH1-R132H, defined tumor-associated peptide panels, or patient-specific neoepitopes; dendritic cell vaccines accounted for ten reports, most loaded with autologous whole tumor lysate; adoptive cell therapies accounted for five (CAR T-EGFRvIII, EGFR-armed bispecific T cells, cytokine-induced killer cells, and ALECSAT); oncolytic viral and gene therapies for three; and autologous formalin-fixed whole tumor vaccines for two. With the single exception of the AFTV trial that predated temozolomide approval in Japan [21], every intervention was added to, rather than substituted for, the Stupp backbone, although temozolomide was omitted in several MGMT-unmethylated protocols. More details can be found in Table 2.

### 3.3. Oncological and Therapeutic Outcomes

Efficacy and safety outcomes are summarized by modality in Table 1 and reported study by study in Table S2. Across the 45 reports, median overall survival ranged from 11.5 to 38.4 months and median progression-free survival from 1.3 to 60.1 months. The highest survival estimates were reported in small, single-arm studies with stringent selection for gross total resection and favorable performance status (Phuphanich et al. 2013 [18], 38.4 months; Batich et al. 2020 [14], 37.7–38.3 months; Cho et al. 2012 [22], 31.9 months; Ji et al. 2018 [23] and Wang et al. 2022 [8], 31.4 months), whereas the lowest were observed in trials restricted to MGMT-unmethylated disease (Bagley et al. 2023 [24], 11.8 months; Lukas et al. 2025 [11], 11.5 months; Lassman et al. 2025 [25], 13 months; Omuro et al. 2023 [26], 13.4 months).

Randomized evidence was consistently negative. None of the five phase III trials, nor the phase II/III trial, demonstrated an OS benefit: nivolumab added to chemoradiotherapy did not improve outcomes in MGMT-methylated or indeterminate disease (28.9 versus 32.1 months [27]) and was inferior to temozolomide in unmethylated disease (13.4 versus 14.9 months [26]); rindopepimut did not prolong survival (20.1 versus 20.0 months [28]); ipilimumab plus nivolumab without temozolomide failed to improve PFS (7.7 versus 8.5 months [25]); and cytokine-induced killer cells improved PFS without an OS gain (8.1 versus 5.4 months [29]), with a positive signal confined to a post hoc pathologically pure GBM subgroup [10]. Among randomized phase II trials, ALECSAT [30], Audencel [31], and nivolumab in elderly patients [32] were likewise negative, and a placebo-controlled phase II trial adding the HSPPC-96 vaccine to pembrolizumab and temozolomide in MGMT-unmethylated disease was discontinued early without benefit (14.4 versus 15.2 months [33]), whereas ICT-107 improved PFS with preserved quality of life (11.2 versus 9.0 months [34]), and dendritic cell vaccination improved short-term survival in a small single-center trial (31.9 versus 15.0 months [22]).

Safety was acceptable across platforms, and no unexpected class-level toxicity emerged. Grade 3 or higher adverse events were largely attributable to the chemoradiotherapy backbone, particularly lymphopenia and thrombocytopenia, and vaccine-based approaches were the best-tolerated, with several trials reporting no grade 3 or higher events at all. The addition of checkpoint inhibitor consistently increased toxicity relative to control arms (52.4% versus 33.6% in Lim et al. 2022 [27]; 46% versus 29% in Sim et al. 2023 [32]), and treatment-related deaths, although rare, occurred almost exclusively in checkpoint inhibitor trials (four in Lim et al. 2022 [27]; three in Omuro et al. 2023 [26]; two in Lassman et al. 2025 [25]). The highest-grade toxicity in the series was seen with cell and gene therapy requiring myeloablative conditioning, where three of twenty-four patients died of conditioning-related infection [35].

Nineteen of the forty-five reports, representing 18 trials, restricted enrollment based on molecular markers (Table S2). MGMT promoter status was used most often: seven admitted only unmethylated disease, one only methylated or indeterminate disease, and one further trial, reported twice, divided its two cohorts on methylation status. HLA type was required in five reports, EGFRvIII expression in three, and IDH1-R132H in one, while the remaining twenty-six enrolled molecularly unselected patients. Enrichment carried no consistent toxicity penalty. Where grade 3 or higher events exceeded those in the control arm, the excess followed the addition of the checkpoint inhibitor rather than the marker used to define the population, and vaccine trials selected on HLA type or antigen expression remained the best-tolerated platforms, whichever marker was applied. Only one trial showed a toxicity difference that mapped onto the biomarker itself: grade 3–4 events reached 75% and grade 5 events reached 33.3% in the unmethylated MGMT cohort, whereas cerebral edema was the sole dose-limiting toxicity in the methylated cohort [11].

Complete data regarding treatment details are presented in Table S2.

**Table 2:** Study design and immunotherapeutic intervention characteristics.

Study (First Author, Year)	Newly Diagnosed GBM	Immunotherapeutic Agent and Modality	Molecular Target	Dose, Schedule, Route, and Duration
Ardon, 2010 [12]	100%	Autologous tumor-lysate-loaded dendritic cell (DC) vaccine	Autologous whole tumor lysate	1–12 × 10^6^ DC per lymph-node region; intradermal; weeks 1–4 (induction), then lysate boosters during maintenance temozolomide; approximately 6 cycles
Ardon, 2012 [13]	100%	Monocyte-derived “early mature” DC loaded with autologous whole tumor lysate	Autologous whole tumor lysate	Intradermal; 4 weekly induction vaccines after chemoradiotherapy, then boosters one week after temozolomide cycles 1, 2, 3, and 6; approximately 6 cycles
Bagley, 2023 [24]	100%	CAR T-EGFRvIII cells (4-1BB/CD3ζ) plus pembrolizumab	EGFRvIII; PD-1	2 × 10^8^ CAR T cells intravenously after hypofractionated radiotherapy (40 Gy/15 fractions), 1 h after pembrolizumab 200 mg; repeated every 3 weeks for up to 4 cycles
Batich, 2020 [14]	100%	Autologous CMV pp65 RNA-pulsed DC vaccine with tetanus–diphtheria toxoid, unpulsed DC or GM-CSF pre-conditioning	Cytomegalovirus pp65	2 × 10^7^ DC intradermally (groin); 3 biweekly doses, then monthly until progression, with standard-dose or dose-intensified temozolomide (≥6 cycles)
Bota, 2022 [36]	100%	AV-GBM-1 (autologous DC loaded with tumor-initiating-cell lysate)	Tumor-initiating-cell antigens	Subcutaneous; 3 weekly doses (weeks 1–3) after chemoradiotherapy, then weeks 8, 12, 16, 20, and 24; up to 8 doses over 24 weeks
Buchroithner, 2018 [31]	100%	Audencel (autologous tumor-lysate-charged, LPS/IFNγ-matured DC)	Autologous whole tumor lysate	1–5 × 10^6^ DC intranodally; 3 weekly doses (weeks 7–10), then weeks 12, 16, 20, 24, and 32, then every 3 months; median 10 doses over 7 months
Bunse, 2026 [16]	100%	IDH1-vac (IDH1-R132H 20-mer peptide) with topical imiquimod	IDH1 R132H neoepitope (MHC class II-restricted)	300 µg peptide in Montanide subcutaneously plus topical imiquimod; 8 doses through week 23; booster doses approximately 6 years later in two participants
Chen, 2025 [37]	100% (13% IDH-mutant)	Tumor Treating Fields plus pembrolizumab plus temozolomide	PD-1; cGAS/STING and AIM2 activation by Tumor Treating Fields	Tumor Treating Fields continuously (≥50% use); temozolomide 150 → 200 mg/m^2^ days 1–5 every 28 days (6–12 cycles); pembrolizumab 200 mg intravenously every 3 weeks from cycle 2; up to 2 years
Cho, 2012 [22]	100%	Autologous whole-tumor-lysate-pulsed DC vaccine	Autologous whole tumor lysate	2–5 × 10^7^ DC/mL subcutaneously (subaxillary); 10 doses over 6 months (weekly ×4, biweekly ×2, then monthly ×4) with chemoradiotherapy and temozolomide
Fadul, 2024 [38]	100% (80% IDH wild type)	EGFR BATs (anti-CD3 × anti-EGFR bispecific antibody-armed autologous T cells)	EGFR and CD3	8 intravenous infusions of 10 × 10^9^ cells; scheme A with adjuvant temozolomide over approximately 7–8 months, scheme B weekly ×8 without temozolomide
Fares, 2021 [20]	92% GBM (100% newly diagnosed high-grade glioma)	NSC-CRAd-S-pk7 (oncolytic adenovirus delivered by allogeneic neural stem cells)	Survivin-promoter-driven tumor-selective viral replication	Single intra-cavitary injection at up to 10 sites (6.25 × 10^10^ to 1.875 × 10^11^ viral particles); chemoradiotherapy started 10–14 days later
Gentner, 2026 [35]	100%	Temferon (autologous TIE2-driven interferon-α gene-modified CD34^+^ hematopoietic stem/progenitor cells)	TIE2-driven interferon-α expression in tumor-associated macrophages	Single intravenous infusion of 0.5–4.0 × 10^6^ CD34^+^ cells/kg after submyeloablative conditioning; radiotherapy 60 Gy without temozolomide
Han, 2022 [10]	100% (80% pathologically pure GBM)	Autologous cytokine-induced killer (CIK) cells	None (MHC-unrestricted CD3^+^CD56^+^ cytotoxicity)	Mean 6.55 × 10^9^ cells intravenously per infusion; 14 infusions (weekly ×4, every 2 weeks ×4, every 4 weeks ×6) added to radiotherapy plus temozolomide
Hilf, 2019 [39]	100%	APVAC1 and APVAC2 personalized peptide and neoantigen vaccines with poly-ICLC and GM-CSF	Unmutated tumor-presented antigens (APVAC1); patient-specific neoepitopes (APVAC2)	400 µg per peptide intradermally; APVAC1 ×11 from day 15 of temozolomide cycle 1; APVAC2 ×8 from cycle 4; median 12 and 10 applications
Inogés, 2017 [40]	100%	Tumor-lysate-pulsed autologous DC vaccine	Autologous whole tumor lysate	10 × 10^6^ DC intradermally; before radiotherapy, 3 weeks after radiotherapy, then 2 monthly, 4 bimonthly, and subsequent quarterly doses; mean 8 vaccines
Ishikawa, 2014 [41]	100%	Autologous formalin-fixed tumor vaccine (AFTV)	None (autologous whole-tumor-derived)	3 courses at 1-week intervals, each of 5 intradermal injections of 0.2 mL; started with temozolomide maintenance cycle 1; approximately 3 weeks
Jacques, 2021 [42]	100%	Avelumab (anti-PD-L1 monoclonal antibody)	PD-L1	10 mg/kg intravenously every 2 weeks from the first post-radiotherapy temozolomide cycle, throughout 6 months of temozolomide and then as monotherapy until progression
Ji, 2018 [23]	100%	HSPPC-96 (autologous heat shock protein–peptide complex 96)	None (autologous tumor-derived peptide repertoire)	25 µg subcutaneously weekly ×6, each preceded by cyclophosphamide 400 mg intravenously; started with adjuvant temozolomide
Johanns, 2024 [17]	100%	NeoVax (multisector-informed personalized neoantigen synthetic long peptide vaccine) with poly-ICLC; nivolumab at progression	Patient-specific HLA class I neoantigens; PD-1	Up to 20 peptides in up to 4 pools subcutaneously with 1.5 mg poly-ICLC; priming on days 1, 8, 15, and 22, then monthly boosters; radiotherapy without temozolomide
Kesari, 2024 [43]	100%	Nivolumab plus ipilimumab in a pre-radiotherapy window	PD-1 and CTLA-4	Nivolumab 300 mg intravenously every 2 weeks and ipilimumab 1 mg/kg every 6 weeks until progression or toxicity; started a median of 38 days after surgery, before chemoradiotherapy
Keskin, 2019 [44]	100%	Personalized multi-epitope neoantigen peptide vaccine with poly-ICLC	Patient-specific somatic neoantigens	Approximately 300 µg per peptide with 0.5 mg poly-ICLC subcutaneously; 5 priming doses over 4 weeks, boosters at weeks 8 and 16; radiotherapy without temozolomide
Kong, 2017 [29]	100%	Autologous cytokine-induced killer (CIK) cells	None (MHC-unrestricted)	Mean 6.55 × 10^9^ cells intravenously over 60 min; 14 infusions (weekly ×4, every 2 weeks ×4, every 4 weeks ×6) over approximately 10 months, added to radiotherapy plus temozolomide
Lassman, 2025 [25]	100%	Ipilimumab plus nivolumab without temozolomide	CTLA-4 and PD-1	Ipilimumab 1 mg/kg intravenously every 4 weeks (maximum 4 doses) and nivolumab 3 mg/kg every 2 weeks (or 240 mg) until progression, with radiotherapy 60 Gy/30 fractions
Lim, 2022 [27]	100%	Nivolumab added to radiotherapy plus temozolomide	PD-1	240 mg intravenously every 2 weeks ×8, then 480 mg every 4 weeks, with radiotherapy 60 Gy/30 fractions, concomitant temozolomide 75 mg/m^2^ and 6 adjuvant cycles; median 10.4 months
Muragaki, 2011 [21]	100%	Autologous formalin-fixed tumor vaccine (AFTV)	None (autologous whole-tumor-derived)	3 courses at 1-week intervals from 32–36 Gy of radiotherapy, each of 5 intradermal injections of 0.2 mL; approximately 3 weeks; no concomitant temozolomide
Omuro, 2022 [45]	100%	Nivolumab with radiotherapy, with or without temozolomide	PD-1	Nivolumab 3 mg/kg intravenously every 2 weeks until progression or toxicity; radiotherapy 60 Gy/30 fractions, with or without temozolomide 75 mg/m^2^ concomitant and 150–200 mg/m^2^ days 1–5 every 28 days for ≥6 cycles
Omuro, 2023 [26]	100%	Nivolumab plus radiotherapy (versus temozolomide plus radiotherapy)	PD-1	Nivolumab 240 mg intravenously every 2 weeks ×8, then 480 mg every 4 weeks until progression or toxicity, with focal radiotherapy 60 Gy/30 fractions; median 22.1 weeks
Ozer, 2026 [33]	100% (all MGMT-unmethylated)	Pembrolizumab plus temozolomide plus HSPPC-96 vaccine or matched placebo	PD-1; autologous tumor peptide repertoire	Pembrolizumab 200 mg intravenously every 3 weeks with radiotherapy 60 Gy and temozolomide; six 9-week adjuvant cycles, then pembrolizumab alone for 12 months; vaccine or placebo intradermally every 4 weeks
Parney, 2022 [46]	100%	Autologous DC pulsed with allogeneic GBM cell-line lysate	Multiple tumor-associated antigens, including EGFRvIII, HER2, gp100, MAGE-A3, and IL13Rα2	2 × 10^7^ DC intradermally; cycles 2–3 on days 1, 3, and 5; cycles 4–6 on day 1 with temozolomide; cycles 7–12 vaccine alone; up to twelve 28-day cycles (~48 weeks)
Phuphanich, 2013 [18]	80% of treated patients	ICT-107 (autologous DC pulsed with 6 synthetic HLA class I peptides)	AIM-2, MAGE1, TRP-2, gp100, HER2/neu, and IL13Rα2	Intradermally in the axilla; 3 doses every 2 weeks (days 0, 14, and 28) after radiotherapy, followed by maintenance temozolomide
Rampling, 2016 [47]	100%	IMA950 multi-peptide vaccine with GM-CSF	11 HLA-A02-restricted tumor-associated peptides	GM-CSF 75 µg followed by IMA950 4.96 mg intradermally at 11 time points over 24 weeks (6 induction, 5 maintenance), with radiotherapy plus temozolomide
Sampson, 2010 [48]	100%	PEPvIII-KLH (EGFRvIII peptide conjugated to keyhole limpet hemocyanin)	EGFRvIII	Intradermally in the inguinal region; 3 doses every 2 weeks from 4 weeks after radiotherapy, then monthly until radiographic progression or death
Schuster, 2015 [49]	100%	Rindopepimut (CDX-110) with GM-CSF	EGFRvIII	500 µg with 150 µg GM-CSF intradermally on days 0, 14, and 28, then monthly with adjuvant temozolomide until intolerance or progression; median 7.4 months
Sim, 2023 [32]	100%	Nivolumab added to temozolomide	PD-1	240 mg intravenously on days 1 and 15 every 28 days (cycles 1–4), then 480 mg on day 1 every 28 days (cycles 5–6), with adjuvant temozolomide after 40 Gy/15 fractions; up to 6 cycles
Sloan, 2024 [50]	100%	Ipilimumab and/or nivolumab with temozolomide	CTLA-4 and/or PD-1	Ipilimumab 3 mg/kg every 4 weeks ×4, then every 3 months ×4; nivolumab 3 mg/kg every 2 weeks ×16; combination cohort ipilimumab 1 mg/kg; all with temozolomide days 1–5 every 28 days ×6
Wainwright, 2025a ‡ [11]	100%	Nivolumab plus BMS-986205 (IDO1 enzyme inhibitor) with radiotherapy, with or without temozolomide	PD-1 and IDO1	BMS-986205 50 or 100 mg (cohort A) or 25 mg (cohort B) orally once daily; nivolumab 240 mg intravenously every 2 weeks, then 480 mg every 4 weeks from cycle 6; radiotherapy 60 Gy over 6 weeks
Wainwright, 2025b ‡ [11]	100%	Nivolumab plus BMS-986205 (IDO1 enzyme inhibitor) with radiotherapy, with or without temozolomide	PD-1 and IDO1	BMS-986205 50 or 100 mg (cohort A) or 25 mg (cohort B) orally once daily; nivolumab 240 mg intravenously every 2 weeks, then 480 mg every 4 weeks from cycle 6; radiotherapy 60 Gy over 6 weeks
Wang, 2022 [8]	100%	HSPPC-96 (autologous gp96–peptide complex vaccine)	Patient-specific tumor peptides chaperoned by gp96	25 µg weekly ×6 following postoperative concurrent chemoradiotherapy; approximately 6 weeks
Weathers, 2025 [51]	100%	Atezolizumab added to radiotherapy plus temozolomide	PD-L1	840 mg intravenously every 2 weeks during the concurrent and adjuvant stages; radiotherapy 60 Gy with temozolomide 75 mg/m^2^ days 1–42, then 150–200 mg/m^2^ days 1–5 every 28 days for up to 12 cycles
Weller, 2017 [28]	100%	Rindopepimut (CDX-110) with GM-CSF versus keyhole limpet hemocyanin control	EGFRvIII	500 µg with 150 µg GM-CSF intradermally on days 1 and 15, then monthly on day 22 of each temozolomide cycle, continued until progression or intolerance
Wen, 2019 [34]	100%	ICT-107 (autologous DC pulsed with 6 peptide epitopes)	MAGE-1 and AIM-2 (HLA-A1); HER2/neu, TRP-2, gp100 and IL13Rα2 (HLA-A2)	1.1 × 10^7^ DC intradermally weekly ×4, then on day 21 of temozolomide cycles 1, 3, 6, and 10, then every 6 months until product depletion or progression
Werlenius, 2021 [30]	100%	ALECSAT (autologous lymphoid effector cells specific against tumor)	Cancer/testis antigens (broad repertoire)	1 × 10^7^ to 1 × 10^9^ cells intravenously every 4 weeks ×3 (loading from week 8), then maintenance every 12 weeks from week 35, with radiotherapy 60 Gy plus temozolomide
Wheeler, 2016 [19]	90% GBM (100% newly diagnosed malignant glioma)	AdV-tk (aglatimagene besadenovec) with valacyclovir	HSV-thymidine kinase/prodrug activation	1 mL injected into up to 10 sites intraoperatively (3 × 10^10^ to 3 × 10^11^ viral particles) plus valacyclovir 2 g orally three times daily for 14 days; then radiotherapy plus temozolomide
Withers, 2025 [15]	100%	SurVaxM (survivin-peptide-conjugate vaccine) with temozolomide	Survivin (BIRC5)	4 subcutaneous priming doses in Montanide ISA-51 every 2 weeks, then maintenance every 3 months with adjuvant temozolomide until progression; median 7.5 doses
Zhang, 2020 [9]	100%	HSPPC-96 (autologous gp96–peptide complex vaccine) with temozolomide	None (autologous tumor-derived)	25 µg subcutaneously weekly ×6, each preceded by cyclophosphamide 400 mg intravenously, concurrent with adjuvant temozolomide

**Abbreviations:** CLIA, Clinical Laboratory Improvement Amendments; CMV, cytomegalovirus; DC, dendritic cell; ECOG, Eastern Cooperative Oncology Group; FFPE, formalin-fixed paraffin-embedded; GBM, glioblastoma; GM-CSF, granulocyte–macrophage colony-stimulating factor; KPS, Karnofsky Performance Status; MGMT, O^6^-methylguanine-DNA methyltransferase; 5-ALA, 5-aminolevulinic acid. ‡ Two separate records extracted from the same trial (NCT04047706). Eligibility criteria previously shown in this table are reported in full in Section 2.2.

## 4. Discussion

Immunotherapy for newly diagnosed GBM is reliably feasible, manufacturable, and tolerable, and frequently induces measurable systemic and intratumoral immune responses, yet these immunological effects have not so far translated into a survival benefit in randomized comparisons. Correlative studies nested within these trials converge on a common explanation, identifying pre-existing tumor immune status, interferon-responsive gene signatures, B-cell infiltration, T-cell receptor repertoire expansion, and antigen expression as candidate determinants of response [8,9,15,18,51].

### 4.1. Immune Checkpoint Inhibitors

Immune checkpoint inhibitors were the most frequently investigated modality in this review, evaluated in 13 of 45 reports and targeting PD-1, PD-L1, CTLA-4, or IDO1. The rationale is that GBM engages coinhibitory axes that terminate T-cell effector function, so antibody blockade should release pre-existing antitumor immunity without requiring identification of a target antigen—a decisive advantage in an antigenically heterogeneous tumor—an assumption reinforced by evidence that neoadjuvant PD-1 blockade upregulates intratumoral T-cell and interferon-γ signatures and confers a survival advantage over adjuvant-only dosing in recurrent disease [52], and by the expectation of synergy with radiotherapy-induced immunogenic cell death, which explains why every included trial added the inhibitor to, rather than substituted it for, the chemoradiotherapy backbone.

This rationale has not translated into benefit: no randomized comparison showed an OS advantage, with nivolumab failing to improve outcomes in MGMT-methylated or indeterminate disease (28.9 versus 32.1 months [27]), proving inferior to temozolomide in unmethylated disease (13.4 versus 14.9 months [26]), and yielding no gain in dual CTLA-4/PD-1 blockade (PFS 7.7 versus 8.5 months [25]) or in elderly patients (11.6 versus 11.8 months [32]), while single-arm experience with avelumab (15.3 months [42]), atezolizumab (18.0 months [51]), and ipilimumab–nivolumab (20.7 months [50]) fell within the range expected from standard care, and outcomes tracked MGMT status far more closely than treatment assignment (33.4 versus 16.5 months by methylation [45]), mirroring the negative phase III experience in recurrent disease [53]. Combination with an antigen-specific platform did not overcome this ceiling either, as the addition of the autologous HSPPC-96 vaccine to pembrolizumab and temozolomide in MGMT-unmethylated disease produced no separation from placebo in either overall or progression-free survival (14.4 versus 15.2 months; 9.9 versus 7.9 months), and the trial was discontinued early [33].

Safety outcomes were reassuring in that no unexpected neurological toxicity emerged, yet grade 3–4 events were consistently more frequent than with control, immune-related events affected approximately a quarter of patients [42], and the few treatment-related deaths in this review occurred almost exclusively in checkpoint inhibitor trials, leaving the efficacy–toxicity balance currently unfavorable. The likely explanations—a low mutational burden and myeloid-dominated, T-cell-poor microenvironment that treatment-induced hypermutation does not rescue [54], molecularly determined response with PTEN alterations enriched in non-responders and MAPK alterations in responders [55], and iatrogenic lymphodepletion with corticosteroid- and surgery-related T-cell sequestration [56]—point to biomarker-enriched selection [57], steroid- and lymphocyte-sparing design, window-of-opportunity dosing [43], and microenvironment-directed combinations as the priority directions, of which only Tumor Treating Fields plus pembrolizumab has so far generated a positive signal (24.8 versus 14.6 months in a case-matched comparison [37]), requiring randomized confirmation.

### 4.2. Peptide and Neoantigen Vaccines

Peptide and neoantigen vaccines were the second most represented modality (12 of 45 reports), targeting EGFRvIII [28,48,49], survivin [15], IDH1-R132H [16], defined HLA-restricted peptide panels [39,47], autologous gp96-peptide complexes [8,9,23], and personalized neoantigen repertoires [17,44]. Their rationale is complementary to checkpoint blockade: rather than releasing pre-existing immunity, vaccination generates it against truly tumor-restricted targets, exploiting the one immunological asset GBM reliably offers while minimizing autoimmune risk. Safety was the best of any platform reviewed, with toxicity largely confined to injection-site reactions. Efficacy was not: single-arm estimates of 15.3–31.4 months were confounded by selection for gross total resection and antigen positivity, and the definitive test was negative, with rindopepimut failing to prolong survival in ACT IV despite robust EGFRvIII-specific antibody responses (20.1 versus 20.0 months [28]).

Two mechanisms explain this dissociation: antigen escape, since EGFRvIII is subclonal [58] and its loss at recurrence was documented in the earliest trial of the series, and an efficacy ceiling imposed by the microenvironment [59], with induced T cells detectable systemically and intratumorally yet universal progression [44]. Priority directions are consequently clonal, driver-derived, and multi-epitope targets that raise the barrier to escape, in combination with checkpoint blockade or myeloid-directed agents rather than further vaccine optimization alone (a strategy whose first randomized test, HSPPC-96 added to pembrolizumab and temozolomide, was stopped early without benefit and with grade 3–4 events in two thirds of patients [33]), accelerated personalized manufacture (mean 164 days, feasibility endpoint missed [17]), and biomarker-guided selection using pre-treatment antigen expression and tumor immune status, both of which correlated with outcome [15,18].

### 4.3. Dendritic Cells Vaccines

Dendritic cell vaccines accounted for 10 of 45 reports and were the earliest immunotherapeutic platform tested [12]. Their rationale differs from peptide vaccination in that antigen selection is bypassed altogether: loading professional antigen-presenting cells with autologous whole tumor lysate delivers a polyvalent, patient-specific antigen repertoire—including unknown and subclonal epitopes—while ex vivo maturation circumvents tumor-induced dendritic cell dysfunction. Safety was excellent and comparable to that of peptide vaccines, with adverse events largely attributable to the chemoradiotherapy backbone; the notable exception was the AV-GBM-1 trial, in which treatment-emergent CNS events were frequent (seizure 12.2%, grade 3 cerebral edema 10.5% [36]). This tolerability was confirmed in the two largest single-arm cohorts, in which grade 3 neurological and hematological events each affected no more than 6.5% of 77 vaccinated patients, with a single treatment-related death (1.3%) [13], and in which severe events represented only 7.9% of all adverse events and were attributed to standard therapy rather than to vaccination [40].

Efficacy, however, remains unproven. Single-arm survival estimates were among the highest in this review (23.4 to 38.3 months), but these came from small, heavily selected cohorts requiring gross total resection, and the two randomized trials in this cohort diverge: the multicenter Audencel trial was negative (18.8 versus 18.9 months [31]), whereas ICT-107 improved PFS with preserved quality of life yet missed its OS endpoint (11.2 versus 9.0 months [34]), and the only positive survival signal came from a small single-center randomized trial (31.9 versus 15.0 months [22]). The external phase III experience is similarly inconclusive, with DCVax-L reporting a survival extension against contemporaneous external rather than randomized controls [60]. The larger single-arm series are themselves sobering in this respect: the HGG-2006 trial, which fully integrated dendritic cell vaccination into postoperative chemoradiotherapy in 77 patients, reached only 18.3 months in the intention-to-treat population, with the survival signal concentrated in the subgroup with minimal residual disease [13], and a phase II trial restricted to complete 5-aminolevulinic acid-guided resection with residual tumor below 1 cm^3^ reported 23.4 months [40].

Priority directions therefore center on the two variables these trials identified as modifiable: enhancing vaccine-site migration and lymph-node homing through pre-conditioning [14], and standardizing manufacture, where allogeneic lysate platforms remove dependence on adequate autologous tumor yield [46]—combined with HLA- and antigen-guided selection, given that activity concentrated in the HLA-A2 subgroup [34] and correlated with pre-vaccine antigen expression [18].

### 4.4. Adoptive Cell Therapies

Adoptive cell therapies were represented by five reports spanning two mechanistically distinct approaches: antigen-redirected products, namely CAR T-EGFRvIII cells [24,61] and anti-CD3 × anti-EGFR bispecific antibody-armed T cells [38]; and non-antigen-specific, MHC-unrestricted effectors, namely cytokine-induced killer cells [10,29] and ALECSAT [30]. The shared rationale is to bypass endogenous priming altogether with the redirected products additionally conferring HLA-independent specificity. Safety was acceptable and manageable, with no treatment-related deaths in any study, although grade ≥ 3 events were common where cells were added to full standard care.

Efficacy was modest and platform-dependent: the phase III cytokine-induced killer trial improved PFS without an OS gain (8.1 versus 5.4 months [29]), with a benefit confined to a post hoc pathologically pure GBM subgroup (23.1 versus 14.9 months [10]); ALECSAT was negative (19.2 versus 18.3 months [30]); and the antigen-redirected products were tested only in very small phase I cohorts, with CAR T-EGFRvIII proving safe and biologically active yet clinically inactive (11.8 months; n = 7 [24]) and EGFR BATs achieving encouraging survival at the maximum feasible dose (28.8 months; n = 10 [38]).

The limiting factors are antigen escape against subclonal EGFRvIII, poor intratumoral trafficking, and persistence after systemic infusion. Future directions accordingly favor locoregional delivery and dual-antigen or T-cell-engager-secreting constructs, an approach that produced rapid radiographic regression after a single intraventricular infusion in recurrent disease, albeit transiently [62], together with lymphodepleting conditioning and adequately powered trials.

### 4.5. Oncolytic Viral, Gene Therapy, and Whole Tumor Vaccine Approaches

Five reports evaluated less-developed platforms whose common rationale is in situ or polyvalent immunization without antigen selection. Three used viral or gene-based strategies—a survivin-promoter-driven oncolytic adenovirus delivered by neural stem cells [20], adenoviral HSV-thymidine kinase with valacyclovir [19], and autologous CD34+ cells engineered for TIE2-restricted interferon-α expression to reprogram tumor-associated macrophages [35]—aiming to convert an immunologically cold microenvironment into an inflamed one. Intratumoral delivery was well tolerated with no dose-limiting toxicities, whereas the gene therapy platform carried the highest toxicity in this review owing to its myeloablative conditioning, and efficacy signals were modest and uncontrolled (18.4 months, Fares et al. 2021 [20]; 16.7 months, Gentner et al. 2026 [35]; 17.1 versus 13.5 months against matched controls, rising to 25.1 versus 16.3 months after gross total resection [19]), with single-dose administration a key constraint that supports the durable responses seen with repeat intratumoral oncolytic herpesvirus [63].

The remaining two trials used autologous formalin-fixed whole tumor vaccine [21,41], a deliberately low-technology approach in which fixation preserves antigenicity and acts as its own adjuvant, requiring no HLA restriction, cell culture, or leukapheresis; it produced the best tolerability of any platform reviewed (no events exceeding grade 1) and encouraging survival (21.4 and 22.2 months), but remains untested in any randomized trial and is arguably the clearest example here of a modality whose development stalled at the feasibility stage rather than having been refuted.

### 4.6. Priorities for Future Trials

The gaps left by these trials are as informative as their results. Older patients were addressed by a single dedicated trial [32]; patients treated by biopsy alone and those unable to taper corticosteroids were excluded by protocol almost throughout; no trial randomized patients between two mechanistically distinct immunotherapeutic platforms, so the relative merit of the six modalities remains unknown; quality of life was seldom reported and immune monitoring was rarely pre-specified with a defined threshold; and no trial randomized the timing of immunotherapy relative to chemoradiation. Therefore, the evidence assembled here supports a small number of specific, testable proposals rather than further unselected testing of existing platforms.

First, randomized trials should be biomarker-enriched at entry rather than analyzed by biomarkers after the fact. Survival tracked MGMT promoter status and extent of resection more closely than treatment assignment across every modality reviewed [45], and the only within-trial toxicity difference attributable to a biomarker arose between the MGMT-defined cohorts of a single protocol [11]. Checkpoint blockade, in particular, warrants a randomized test restricted to patients with an inflamed pre-treatment tumor-immune phenotype, defined by interferon-responsive gene expression, B-cell infiltration, or T-cell receptor repertoire expansion, each of which is correlated with outcomes in the correlative analyses nested within the trials reviewed here [8,9,15,51].

Second, the trial design should protect the effector compartment on which these therapies depend. Every included protocol added immunotherapy to a chemoradiotherapy backbone that reliably produces lymphopenia, which is compounded by corticosteroid exposure and surgical trauma [56]. Lymphocyte-sparing radiotherapy, strict corticosteroid ceilings, and randomization of the timing of immunotherapy relative to chemoradiation are each testable in the first-line setting, but none has been tested.

Third, dosing should be performed earlier. Neoadjuvant PD-1 blockade before resection conferred a survival advantage over adjuvant-only dosing in recurrent disease [52], and a pre-radiotherapy window was proven feasible in newly diagnosed patients [43]; however, no trial in this review randomized neoadjuvant against adjuvant scheduling. A window-of-opportunity trial allocating patients to pre-surgical or post-surgical initiation, with paired tumor tissue as the primary endpoint, would establish whether the observation in recurrent disease generalizes to the first-line setting.

Fourth, antigen-directed platforms require targets that can survive the selection pressure. EGFRvIII vaccination reproducibly generates the intended immune response and reproducibly fails because the target is subclonal and is lost at the time of recurrence [28,48,58]. Multi-epitope constructs built on clonal, driver-derived antigens and locoregional rather than systemic delivery of redirected T cells are the two modifications with a mechanistic rationale; intraventricular delivery of a dual-targeting construct produces rapid radiographic regression in recurrent disease and merits a first-line therapy [62].

Fifth, two platforms in this review stalled before they were tested rather than after they had failed. Autologous formalin-fixed tumor vaccines produced the best tolerability of any modality reviewed and encouraging uncontrolled survival, yet have never entered a randomized trial [21,41]. Tumor Treating Fields combined with pembrolizumab produced the only positive signal among checkpoint inhibitor combinations against a case-matched control [37]. Both are ready for adequately powered randomized confirmation.

Finally, the reporting deficiencies documented in this study should be corrected prospectively. Future first-line immunotherapy trials should be registered before enrolment, report responses against a single contemporary standard, state MGMT and IDH status for every participant, report adverse events per patient rather than per event, and pre-specify the biomarker hypothesis they intend to test.

### 4.7. Limitations

Several limitations qualify these findings. The evidence base is dominated by small, single-arm, early-phase studies with survival estimates frequently derived from cohorts selected for gross total resection and favorable performance status and benchmarked against historical or externally matched controls rather than concurrent randomized arms. Heterogeneity in platform, population, outcome definition, and adverse event reporting precluded meta-analysis; therefore, the evidence was synthesized descriptively, and no pooled effect estimate was presented. A formal risk of bias assessment was not performed—this was pre-specified in the registered protocol and follows JBI guidance, under which critical appraisal is optional in a scoping review, and PRISMA-ScR item 12, which requires the decision to be stated rather than performed; the main reason is that no pooled estimate is reported, which such an assessment would qualify. The design features bearing most directly on interpretability, namely randomization, blinding, use of historical or external controls, and centrally confirmed eligibility, are instead charted for every study in Table S1 and are weighed throughout the synthesis. This remains a limitation, and the survival estimates reported here should be considered descriptive rather than comparative. The period spanned by the included trials crossed successive WHO classifications, meaning that populations labeled GBM are not biologically equivalent across studies. Finally, companion publications from shared trials are reported as separate records, although the reconciliation in Section 3.2 identifies each companion set, and restriction to published reports leaves the review susceptible to publication bias favoring positive early-phase signals.

## 5. Conclusions

Immunotherapy for newly diagnosed GBM is feasible, tolerable, and consistently capable of inducing measurable systemic and intratumoral immune responses. However, it has not improved survival in randomized comparisons, with outcomes remaining more strongly determined by MGMT promoter status and extent of resection than by treatment assignment. These findings, together with the immunosuppressive tumor microenvironment and treatment-related lymphodepletion, suggest that unselected immunotherapy is unlikely to provide meaningful benefit. Future progress will require biomarker-enriched patient selection, lymphocyte- and steroid-sparing trial designs, earlier intervention, and rational combinations evaluated in adequately powered randomized trials.

## Figures and Tables

**Table 1:** Summary of the evidence by immunotherapeutic modality.

Immunotherapeutic Modality	Reports (Trials)	Phase Distribution	Median OS, Months	Median PFS, Months	Grade ≥ 3 Adverse Events	Randomized Evidence
Immune checkpoint inhibitors	13 (12)	I, 5; I/II, 1; II, 4; II/III, 1; III, 2	11.5–33.4	1.3–16.1	Grade ≥ 3 events 7.5–75%, consistently higher than in control arms; treatment-related deaths in three trials (2 to 4 patients each)	Six randomized comparisons, none showing an OS benefit
Peptide and neoantigen vaccines	12 (10)	I, 7; II, 3; III, 1; not reported, 1	15.3–31.4 (not reached in 2)	7.6–60.1	Grade ≥ 3 events 0–68.8%, largely injection-site reactions and temozolomide-related myelosuppression; one treatment-related death	One phase III comparison (ACT IV), negative
Dendritic cell vaccines	10 (10)	I, 2; I/II, 2; II, 6	16.0–38.4	6.8–18.0	Grade ≥ 3 events 0–35%, mostly attributable to the chemoradiotherapy backbone; the exception was AV-GBM-1 (seizure 12.2%, grade 3 cerebral oedema 10.5%); one treatment-related death (1.3%)	Four randomized comparisons, one positive for OS (single center, n = 34)
Adoptive cell therapies	5 (4)	I, 2; II, 1; III, 2	11.8–28.8	5.2–17.2	Grade ≥ 3 events 1.2–92.5%, highest where cells were added to full standard care; no treatment-related deaths	Three randomized comparisons, none showing an OS benefit; PFS gain without OS gain in the phase III cytokine-induced killer trial
Oncolytic viral and gene therapies	3 (3)	I, 1; I/II, 1; Ib/II, 1	16.7–18.4	8.1–9.1	Intratumoral delivery without dose-limiting toxicity; the gene therapy platform carried the highest toxicity in this review, with 3 of 24 patients (12.5%) dying of conditioning-related infection	None
Autologous formalin-fixed tumor vaccine	2 (2)	I/II, 2	21.4–22.2	7.6–8.2	The best tolerability of any platform; no event exceeding grade 1 in one trial, and grade 3–4 events attributed to chemoradiotherapy in the other	None
**Total**	45 (41)	I, 17; I/II, 6; Ib/II, 1; II, 14; II/III, 1; III, 5; not reported, 1	11.5–38.4	1.3–60.1	—	Fourteen randomized comparisons, one positive for OS

Ranges are of the intervention-arm median reported in each study. Trial counts in parentheses treat companion publications of the same trial as a single trial (Section 3.2). Full study-level data are given in Tables S1 and S2. Abbreviations: AE, adverse event; OS, overall survival; PFS, progression-free survival.

## Data Availability

All generated data are presented in the manuscript.

## Supplementary Materials

The following supporting information can be downloaded at: https://www.mdpi.com/article/10.3390/jpm16090479/s1, Table S1. Characteristics of the included studies. Table S2. Survival outcomes, safety and synthesis of findings.

## References

[1] Stupp R., Mason W.P., Van Den Bent M.J., Weller M., Fisher B., Taphoorn M.J.B., Belanger K., Brandes A.A., Marosi C., Bogdahn U. (2005). Radiotherapy plus Concomitant and Adjuvant Temozolomide for Glioblastoma. N. Engl. J. Med..

[2] Putra M.A.R., Ghozali S.A.S., Annisa M.F. (2026). Focused Ultrasound–Induced Blood–Brain Barrier Modulation for Drug Delivery in Recurrent Glioblastoma: A Systematic Review. Indian J. Med. Res..

[3] Lim M., Xia Y., Bettegowda C., Weller M. (2018). Current State of Immunotherapy for Glioblastoma. Nat. Rev. Clin. Oncol..

[4] Angom R.S., Nakka N.M.R., Bhattacharya S. (2023). Advances in Glioblastoma Therapy: An Update on Current Approaches. Brain Sci..

[5] Agosti E., Zeppieri M., De Maria L., Tedeschi C., Fontanella M.M., Panciani P.P., Ius T. (2023). Glioblastoma Immunotherapy: A Systematic Review of the Present Strategies and Prospects for Advancements. Int. J. Mol. Sci..

[6] Aromataris E., Lockwood C., Porritt K., Pilla B., Jordan Z. (2024). JBI Manual for Evidence Synthesis.

[7] Tricco A.C., Lillie E., Zarin W., O’Brien K.K., Colquhoun H., Levac D., Moher D., Peters M.D.J., Horsley T., Weeks L. (2018). PRISMA Extension for Scoping Reviews (PRISMA-ScR): Checklist and Explanation. Ann. Intern. Med..

[8] Wang Y., Li C., Chi X., Huang X., Gao H., Ji N., Zhang Y. (2022). Low MxA Expression Predicts Better Immunotherapeutic Outcomes in Glioblastoma Patients Receiving Heat Shock Protein Peptide Complex 96 Vaccination. Front. Oncol..

[9] Zhang Y., Mudgal P., Wang L., Wu H., Huang N., Alexander P.B., Gao Z., Ji N., Li Q.-J. (2020). T Cell Receptor Repertoire as a Prognosis Marker for Heat Shock Protein Peptide Complex-96 Vaccine Trial against Newly Diagnosed Glioblastoma. OncoImmunology.

[10] Han M.-H., Kim J.M., Cheong J.H., Ryu J.I., Won Y.D., Nam G.H., Kim C.H. (2022). Efficacy of Cytokine-Induced Killer Cell Immunotherapy for Patients With Pathologically Pure Glioblastoma. Front. Oncol..

[11] Lukas R.V., Zhai L., Lauing K.L., Kim M., Koch T., Penco-Campillo M., Bommi P., Dixit K., Kumthekar P., Sharp L. (2026). Phase I Evaluation of Patients with Newly Diagnosed Glioblastoma Treated with Radiation, Nivolumab, and IDO1 Enzyme Inhibitor BMS-986205. Clin. Cancer Res..

[12] Ardon H., Van Gool S., Lopes I.S., Maes W., Sciot R., Wilms G., Demaerel P., Bijttebier P., Claes L., Goffin J. (2010). Integration of Autologous Dendritic Cell-Based Immunotherapy in the Primary Treatment for Patients with Newly Diagnosed Glioblastoma Multiforme: A Pilot Study. J. Neuro-Oncol..

[13] Ardon H., Van Gool S.W., Verschuere T., Maes W., Fieuws S., Sciot R., Wilms G., Demaerel P., Goffin J., Van Calenbergh F. (2012). Integration of Autologous Dendritic Cell-Based Immunotherapy in the Standard of Care Treatment for Patients with Newly Diagnosed Glioblastoma: Results of the HGG-2006 Phase I/II Trial. Cancer Immunol. Immunother..

[14] Batich K.A., Mitchell D.A., Healy P., Herndon J.E., Sampson J.H. (2020). Once, Twice, Three Times a Finding: Reproducibility of Dendritic Cell Vaccine Trials Targeting Cytomegalovirus in Glioblastoma. Clin. Cancer Res..

[15] Withers H.G., Figel S.A., Qiu J., Ahluwalia M.S., Reardon D., Abad A.P., Curry W.T., Wong E.T., Peereboom D.M., Dhawan A. (2025). Intratumoral B Cell and Interferon Signatures in Newly Diagnosed Glioblastoma Are Associated with Longer Survival in Patients Treated with SurVaxM. Cancer Immunol. Immunother..

[16] Bunse L., Lindner K., Wick A., Freitag A., Lanz L.-M., Edelmann D., Suwala A., Breckwoldt M.O., Harting I., Sahm F. (2026). IDH1-Mutant Vaccine in Newly Diagnosed Astrocytoma: Final Analysis of the Multicenter, Single-Arm, Open-Label, First-in-Human Phase 1 NOA16 Trial. Nat. Cancer.

[17] Johanns T.M., Garfinkle E.A.R., Miller K.E., Livingstone A.J., Roberts K.F., Rao Venkata L.P., Dowling J.L., Chicoine M.R., Dacey R.G., Zipfel G.J. (2024). Integrating Multisector Molecular Characterization into Personalized Peptide Vaccine Design for Patients with Newly Diagnosed Glioblastoma. Clin. Cancer Res..

[18] Phuphanich S., Wheeler C.J., Rudnick J.D., Mazer M., Wang H., Nuño M.A., Richardson J.E., Fan X., Ji J., Chu R.M. (2013). Phase I Trial of a Multi-Epitope-Pulsed Dendritic Cell Vaccine for Patients with Newly Diagnosed Glioblastoma. Cancer Immunol. Immunother..

[19] Wheeler L.A., Manzanera A.G., Bell S.D., Cavaliere R., McGregor J.M., Grecula J.C., Newton H.B., Lo S.S., Badie B., Portnow J. (2016). Phase II Multicenter Study of Gene-Mediated Cytotoxic Immunotherapy as Adjuvant to Surgical Resection for Newly Diagnosed Malignant Glioma. Neuro Oncol..

[20] Fares J., Ahmed A.U., Ulasov I.V., Sonabend A.M., Miska J., Lee-Chang C., Balyasnikova I.V., Chandler J.P., Portnow J., Tate M.C. (2021). Neural Stem Cell Delivery of an Oncolytic Adenovirus in Newly Diagnosed Malignant Glioma: A First-in-Human, Phase 1, Dose-Escalation Trial. Lancet Oncol..

[21] Muragaki Y., Maruyama T., Iseki H., Tanaka M., Shinohara C., Takakura K., Tsuboi K., Yamamoto T., Matsumura A., Matsutani M. (2011). Phase I/IIa Trial of Autologous Formalin-Fixed Tumor Vaccine Concomitant with Fractionated Radiotherapy for Newly Diagnosed Glioblastoma: Clinical Article. J. Neurosurg..

[22] Cho D.-Y., Yang W.-K., Lee H.-C., Hsu D.-M., Lin H.-L., Lin S.-Z., Chen C.-C., Harn H.-J., Liu C.-L., Lee W.-Y. (2012). Adjuvant Immunotherapy with Whole-Cell Lysate Dendritic Cells Vaccine for Glioblastoma Multiforme: A Phase II Clinical Trial. World Neurosurg..

[23] Ji N., Zhang Y., Liu Y., Xie J., Wang Y., Hao S., Gao Z. (2018). Heat Shock Protein Peptide Complex-96 Vaccination for Newly Diagnosed Glioblastoma: A Phase I, Single-Arm Trial. JCI Insight.

[24] Bagley S.J., Binder Z.A., Lamrani L., Marinari E., Desai A.S., Nasrallah M.P., Maloney E., Brem S., Lustig R.A., Kurtz G. (2024). Repeated Peripheral Infusions of Anti-EGFRvIII CAR T Cells in Combination with Pembrolizumab Show No Efficacy in Glioblastoma: A Phase 1 Trial. Nat. Cancer.

[25] Lassman A.B., Polley M.-Y.C., Iwamoto F.M., Sloan A.E., Wang T.J.C., Aldape K.D., Wefel J.S., Gondi V., Gutierrez A.N., Manasawala M.H. (2025). Dual Immune Check Point Blockade in *MGMT* -Unmethylated Newly Diagnosed Glioblastoma: NRG Oncology BN007, a Randomized Phase II/III Clinical Trial. J. Clin. Oncol..

[26] Omuro A., Brandes A.A., Carpentier A.F., Idbaih A., Reardon D.A., Cloughesy T., Sumrall A., Baehring J., Van Den Bent M., Bähr O. (2023). Radiotherapy Combined with Nivolumab or Temozolomide for Newly Diagnosed Glioblastoma with Unmethylated *MGMT* Promoter: An International Randomized Phase III Trial. Neuro Oncol..

[27] Lim M., Weller M., Idbaih A., Steinbach J., Finocchiaro G., Raval R.R., Ansstas G., Baehring J., Taylor J.W., Honnorat J. (2022). Phase III Trial of Chemoradiotherapy with Temozolomide plus Nivolumab or Placebo for Newly Diagnosed Glioblastoma with Methylated *MGMT* Promoter. Neuro Oncol..

[28] Weller M., Butowski N., Tran D.D., Recht L.D., Lim M., Hirte H., Ashby L., Mechtler L., Goldlust S.A., Iwamoto F. (2017). Rindopepimut with Temozolomide for Patients with Newly Diagnosed, EGFRvIII-Expressing Glioblastoma (ACT IV): A Randomised, Double-Blind, International Phase 3 Trial. Lancet Oncol..

[29] Kong D.-S., Nam D.-H., Kang S.-H., Lee J.W., Chang J.-H., Kim J.-H., Lim Y.-J., Koh Y.-C., Chung Y.-G., Kim J.-M. (2017). Phase III Randomized Trial of Autologous Cytokine-Induced Killer Cell Immunotherapy for Newly Diagnosed Glioblastoma in Korea. Oncotarget.

[30] Werlenius K., Stragliotto G., Strandeus M., Blomstrand M., Carén H., Jakola A.S., Rydenhag B., Dyregaard D., Dzhandzhugazyan K.N., Kirkin A.F. (2021). A Randomized Phase II Trial of Efficacy and Safety of the Immunotherapy ALECSAT as an Adjunct to Radiotherapy and Temozolomide for Newly Diagnosed Glioblastoma. Neuro-Oncol. Adv..

[31] Buchroithner J., Erhart F., Pichler J., Widhalm G., Preusser M., Stockhammer G., Nowosielski M., Iglseder S., Freyschlag C.F., Oberndorfer S. (2018). Audencel Immunotherapy Based on Dendritic Cells Has No Effect on Overall and Progression-Free Survival in Newly Diagnosed Glioblastoma: A Phase II Randomized Trial. Cancers.

[32] Sim H.-W., Wachsmuth L., Barnes E.H., Yip S., Koh E.-S., Hall M., Jennens R., Ashley D.M., Verhaak R.G., Heimberger A.B. (2023). NUTMEG: A Randomized Phase II Study of Nivolumab and Temozolomide versus Temozolomide Alone in Newly Diagnosed Older Patients with Glioblastoma. Neuro-Oncol. Adv..

[33] Ozer B.H., Lindhorst S.M., Merrell R.T., Trevino C.R., Rudnick J.D., Avgeropoulos N.G., Ramakrishna N., Khagi S., Rauf Y., Walbert T. (2026). Pembrolizumab, Temozolomide and HSPPC-96 Vaccine in Newly Diagnosed Glioblastoma Post-Chemoradiation: Results from a Multi-Institutional, Phase 2, Randomized, Placebo-Controlled Trial. medRxiv.

[34] Wen P.Y., Reardon D.A., Armstrong T.S., Phuphanich S., Aiken R.D., Landolfi J.C., Curry W.T., Zhu J.-J., Glantz M., Peereboom D.M. (2019). A Randomized Double-Blind Placebo-Controlled Phase II Trial of Dendritic Cell Vaccine ICT-107 in Newly Diagnosed Patients with Glioblastoma. Clin. Cancer Res..

[35] Gentner B., Eoli M., Farina F., Barcella M., Capotondo A., Mazzoleni S., Brambilla V., Francavilla A., Garramone M., Carrabba M.G. (2026). Tumor-Targeted Interferon-α Gene Therapy for Glioblastoma: A Phase 1 Trial. Nat. Med..

[36] Bota D.A., Taylor T.H., Piccioni D.E., Duma C.M., LaRocca R.V., Kesari S., Carrillo J.A., Abedi M., Aiken R.D., Hsu F.P.K. (2022). Phase 2 Study of AV-GBM-1 (a Tumor-Initiating Cell Targeted Dendritic Cell Vaccine) in Newly Diagnosed Glioblastoma Patients: Safety and Efficacy Assessment. J. Exp. Clin. Cancer Res..

[37] Chen D., Le S.B., Ghiaseddin A.P., Manektalia H., Li M., O’Dell A., Rahman M., Tran D.D. (2025). Efficacy and Safety of Adjuvant TTFields plus Pembrolizumab and Temozolomide in Newly Diagnosed Glioblastoma: A Phase 2 Study. Med.

[38] Fadul C.E., Thakur A., Kim J., Kassay-McAllister J., Schalk D., Lopes M.B., Donahue J., Purow B., Dillon P., Le T. (2024). Phase I Study Targeting Newly Diagnosed Grade 4 Astrocytoma with Bispecific Antibody Armed T Cells (EGFR BATs) in Combination with Radiation and Temozolomide. J. Neuro-Oncol..

[39] Hilf N., Kuttruff-Coqui S., Frenzel K., Bukur V., Stevanović S., Gouttefangeas C., Platten M., Tabatabai G., Dutoit V., Van Der Burg S.H. (2019). Actively Personalized Vaccination Trial for Newly Diagnosed Glioblastoma. Nature.

[40] Inogés S., Tejada S., De Cerio A.L.-D., Gállego Pérez-Larraya J., Espinós J., Idoate M.A., Domínguez P.D., De Eulate R.G., Aristu J., Bendandi M. (2017). A Phase II Trial of Autologous Dendritic Cell Vaccination and Radiochemotherapy Following Fluorescence-Guided Surgery in Newly Diagnosed Glioblastoma Patients. J. Transl. Med..

[41] Ishikawa E., Muragaki Y., Yamamoto T., Maruyama T., Tsuboi K., Ikuta S., Hashimoto K., Uemae Y., Ishihara T., Matsuda M. (2014). Phase I/IIa Trial of Fractionated Radiotherapy, Temozolomide, and Autologous Formalin-Fixed Tumor Vaccine for Newly Diagnosed Glioblastoma: Clinical Article. J. Neurosurg..

[42] Jacques F.H., Nicholas G., Lorimer I.A.J., Sikati Foko V., Prevost J., Dumais N., Milne K., Nelson B.H., Woulfe J., Jansen G. (2021). Avelumab in Newly Diagnosed Glioblastoma. Neuro-Oncol. Adv..

[43] Kesari S., Wojcinski A., Pabla S., Seager R.J., Gill J.M., Carrillo J.A., Wagle N., Park D.J., Nguyen M., Truong J. (2024). Pre-Radiation Nivolumab plus Ipilimumab in Patients with Newly Diagnosed High-Grade Gliomas. OncoImmunology.

[44] Keskin D.B., Anandappa A.J., Sun J., Tirosh I., Mathewson N.D., Li S., Oliveira G., Giobbie-Hurder A., Felt K., Gjini E. (2019). Neoantigen Vaccine Generates Intratumoral T Cell Responses in Phase Ib Glioblastoma Trial. Nature.

[45] Omuro A., Reardon D.A., Sampson J.H., Baehring J., Sahebjam S., Cloughesy T.F., Chalamandaris A.-G., Potter V., Butowski N., Lim M. (2022). Nivolumab plus Radiotherapy with or without Temozolomide in Newly Diagnosed Glioblastoma: Results from Exploratory Phase I Cohorts of CheckMate 143. Neuro-Oncol. Adv..

[46] Parney I.F., Anderson S.K., Gustafson M.P., Steinmetz S., Peterson T.E., Kroneman T.N., Raghunathan A., O’Neill B.P., Buckner J.C., Solseth M. (2022). Phase I Trial of Adjuvant Mature Autologous Dendritic Cell/Allogeneic Tumor Lysate Vaccines in Combination with Temozolomide in Newly Diagnosed Glioblastoma. Neuro-Oncol. Adv..

[47] Rampling R., Peoples S., Mulholland P.J., James A., Al-Salihi O., Twelves C.J., McBain C., Jefferies S., Jackson A., Stewart W. (2016). A Cancer Research UK First Time in Human Phase I Trial of IMA950 (Novel Multipeptide Therapeutic Vaccine) in Patients with Newly Diagnosed Glioblastoma. Clin. Cancer Res..

[48] Sampson J.H., Heimberger A.B., Archer G.E., Aldape K.D., Friedman A.H., Friedman H.S., Gilbert M.R., Herndon J.E., McLendon R.E., Mitchell D.A. (2010). Immunologic Escape After Prolonged Progression-Free Survival With Epidermal Growth Factor Receptor Variant III Peptide Vaccination in Patients With Newly Diagnosed Glioblastoma. J. Clin. Oncol..

[49] Schuster J., Lai R.K., Recht L.D., Reardon D.A., Paleologos N.A., Groves M.D., Mrugala M.M., Jensen R., Baehring J.M., Sloan A. (2015). A Phase II, Multicenter Trial of Rindopepimut (CDX-110) in Newly Diagnosed Glioblastoma: The ACT III Study. Neuro-Oncol..

[50] Sloan A.E., Winter K., Gilbert M.R., Aldape K., Choi S., Wen P.Y., Butowski N., Iwamoto F.M., Raval R.R., Voloschin A.D. (2024). NRG-BN002: Phase I Study of Ipilimumab, Nivolumab, and the Combination in Patients with Newly Diagnosed Glioblastoma. Neuro-Oncol..

[51] Weathers S.-P., Li X., Zhu H., Damania A.V., Knafl M., McKinley B., Lin H., Harrison R.A., Majd N.K., O’Brien B.J. (2025). Improved Overall Survival in an Anti-PD-L1 Treated Cohort of Newly Diagnosed Glioblastoma Patients Is Associated with Distinct Immune, Mutation, and Gut Microbiome Features: A Single Arm Prospective Phase I/II Trial. Nat. Commun..

[52] Cloughesy T.F., Mochizuki A.Y., Orpilla J.R., Hugo W., Lee A.H., Davidson T.B., Wang A.C., Ellingson B.M., Rytlewski J.A., Sanders C.M. (2019). Neoadjuvant Anti-PD-1 Immunotherapy Promotes a Survival Benefit with Intratumoral and Systemic Immune Responses in Recurrent Glioblastoma. Nat. Med..

[53] Reardon D.A., Brandes A.A., Omuro A., Mulholland P., Lim M., Wick A., Baehring J., Ahluwalia M.S., Roth P., Bähr O. (2020). Effect of Nivolumab vs Bevacizumab in Patients With Recurrent Glioblastoma: The CheckMate 143 Phase 3 Randomized Clinical Trial. JAMA Oncol..

[54] Touat M., Li Y.Y., Boynton A.N., Spurr L.F., Iorgulescu J.B., Bohrson C.L., Cortes-Ciriano I., Birzu C., Geduldig J.E., Pelton K. (2020). Mechanisms and Therapeutic Implications of Hypermutation in Gliomas. Nature.

[55] Zhao J., Chen A.X., Gartrell R.D., Silverman A.M., Aparicio L., Chu T., Bordbar D., Shan D., Samanamud J., Mahajan A. (2019). Immune and Genomic Correlates of Response to Anti-PD-1 Immunotherapy in Glioblastoma. Nat. Med..

[56] Otvos B., Alban T.J., Grabowski M.M., Bayik D., Mulkearns-Hubert E.E., Radivoyevitch T., Rabljenovic A., Johnson S., Androjna C., Mohammadi A.M. (2021). Preclinical Modeling of Surgery and Steroid Therapy for Glioblastoma Reveals Changes in Immunophenotype That Are Associated with Tumor Growth and Outcome. Clin. Cancer Res..

[57] Agosti E., Mapelli K., Grimod G., Piazza A., Fontanella M.M., Panciani P.P. (2026). MRI-Based Radiomics for Non-Invasive Prediction of Molecular Biomarkers in Gliomas. Cancers.

[58] Agosti E., Antonietti S., Ius T., Fontanella M.M., Zeppieri M., Panciani P.P. (2024). Glioma Stem Cells as Promoter of Glioma Progression: A Systematic Review of Molecular Pathways and Targeted Therapies. Int. J. Mol. Sci..

[59] Agosti E., Panciani P.P., Zeppieri M., De Maria L., Pasqualetti F., Tel A., Zanin L., Fontanella M.M., Ius T. (2023). Tumor Microenvironment and Glioblastoma Cell Interplay as Promoters of Therapeutic Resistance. Biology.

[60] Liau L.M., Ashkan K., Brem S., Campian J.L., Trusheim J.E., Iwamoto F.M., Tran D.D., Ansstas G., Cobbs C.S., Heth J.A. (2023). Association of Autologous Tumor Lysate-Loaded Dendritic Cell Vaccination With Extension of Survival Among Patients With Newly Diagnosed and Recurrent Glioblastoma: A Phase 3 Prospective Externally Controlled Cohort Trial. JAMA Oncol..

[61] Agosti E., Garaba A., Antonietti S., Ius T., Fontanella M.M., Zeppieri M., Panciani P.P. (2024). CAR-T Cells Therapy in Glioblastoma: A Systematic Review on Molecular Targets and Treatment Strategies. Int. J. Mol. Sci..

[62] Choi B.D., Gerstner E.R., Frigault M.J., Leick M.B., Mount C.W., Balaj L., Nikiforow S., Carter B.S., Curry W.T., Gallagher K. (2024). Intraventricular CARv3-TEAM-E T Cells in Recurrent Glioblastoma. N. Engl. J. Med..

[63] Todo T., Ino Y., Ohtsu H., Shibahara J., Tanaka M. (2022). A Phase I/II Study of Triple-Mutated Oncolytic Herpes Virus G47∆ in Patients with Progressive Glioblastoma. Nat. Commun..

