# Supplementary material for: Immunotherapy for Newly Diagnosed Glioblastoma: Current Evidence and Future Perspectives
Source: J Pers Med. 2026 Sep 17;16(9):479. doi: 10.3390/jpm16090479 (PMC13608823; doi:10.3390/jpm16090479)
Supplement: Supplementary file 1 [file jpm-16-00479-s001.zip › jpm-4523339-supplementary tables.pdf]

Supplementary Materials

Table S1. Characteristics of the included studies.

Study (first author, year)	Country	SC/MC (n centres)	Phase	Trial design and comparator	Blinding	Sample size	Median age, years	Female, n (%)
Ardon, 2010 [53]	Belgium	SC (1)	I/II	Single-arm, non-randomised; no comparator	NR	8 treated	50 (31–62)	3 (37.5)
Ardon, 2012 [55]	Belgium	SC (1)	I/II	Single-arm, non-randomised; no comparator	NR	77 treated	57 (26–70)	29 (37.7)
Bagley, 2023 [16]	USA, France, Switzerland	SC (1)	I	Single-arm dose-finding; no comparator	Open label	7 treated	NR (range 56–76)	2 (28.6)
Batich, 2020 [12]	USA	SC (1)	II	Randomised pre-conditioning sub-study plus single-arm cohort; historical SOC controls	Blinded (ATTAC); open (ATTAC-GM)	23 treated (+23 historical controls)	50.5–65 by cohort	10 (43.5)
Bota, 2022 [54]	USA	MC (7)	II	Single-arm; no comparator	Open label	60 enrolled / 57 treated	59 (29–70)	18 (30.0)
Buchroithner, 2018 [25]	Austria	MC (9)	II	Randomised 1:1 versus standard of care alone	Open label	76 analyzed (34/42)	54	25 (32.9)
Bunse, 2026 [46]	Germany	MC (8)	I	Single-arm; historical cohorts for context	Open label	32 treated	40.4 ± 9.0*	12 (37.5)
Chen, 2025 [43]	USA	SC (1–2)	II	Single-arm; case-matched EF-14 historical control (n = 56)	NR	31 ITT / 28 treated	60.7	9 (29.0)
Cho, 2012 [13]	Taiwan	SC (1)	II	Randomised versus surgery, radiotherapy and chemotherapy	Open label (not blindable)	34 randomised (18/16)	58 versus 58.5	18 (52.9)
Fadul, 2024 [60]	USA	SC (1)	I	Single-arm dose-escalation plus expansion; no comparator	NR	10 treated	NR	6 (60.0)
Fares, 2021 [10]	USA	MC (2)	I	Single-arm 3+3 dose-escalation	Open label	12 treated	52 (IQR 48–65)	7 (58.3)
Gentner, 2026 [29]	Italy	MC (4)	I/II	Single-arm dose-escalation (8 cohorts)	Open label	24 dosed (27 ITT)	59 (29–67)	9 (37.5)
Han, 2022 [23]	South Korea	MC (7)	III	Randomised versus temozolomide chemoradiotherapy alone (pure-GBM subgroup)	Open label	125 pure GBM (156 mITT)	53.3 ± 10.4*	68 (43.6)†
Hilf, 2019 [48]	Germany, Switzerland, Netherlands, Spain, Denmark	MC (6)	I	Single-arm; no comparator	Open label	16 enrolled / 15 vaccinated	52.5 (25–70)	7 (43.8)
Inogés, 2017 [56]	Spain	SC (1)	II	Single-arm; no comparator	NR	31 ITT / 29 treated	61 (42–70)	15 (48.4)
Ishikawa, 2014 [63]	Japan	MC (3)	I/II	Single-arm; no comparator	Open label	24 analyzed (26 enrolled)	48 (26–66)*	7 (29.2)
Jacques, 2021 [34]	Canada	SC (1)	II	Single-arm; no comparator	Open label	30 treated	55 (31–74)	13 (43.3)
Ji, 2018 [14]	China	SC (1)	I	Single-arm; no comparator	Open label	20 treated	52 (40–70)	10 (50.0)
Johanns, 2024 [50]	USA	SC (1)	NR (pilot)	Single-arm feasibility; no comparator	NR	4 enrolled / 3 vaccinated	NR (range 51–62)	2 (50.0)

Study (first author, year)	Country	SC/MC (n centres)	Phase	Trial design and comparator	Blinding	Sample size	Median age, years	Female, n (%)
Kesari, 2024 [42]	USA	SC (1)	I	Single-arm; no comparator	NR	15 treated	66 (39–78)	4 (26.7)
Keskin, 2019 [49]	USA	SC (1)	I	Single-arm; no comparator	NR	10 enrolled / 8 vaccinated	NR	NR
Kong, 2017 [22]	South Korea	MC (7)	III	Randomised versus temozolomide alone	Open label	180 ITT (91/89)	55 versus 54	78 (43.3)
Lassman, 2025 [18]	USA	MC (NRG network)	II/III	Randomised versus radiotherapy plus temozolomide	Open label	159 randomised (79/80)	60 (28–79)	54 (34.0)
Lim, 2022 [20]	International (19 countries)	MC (118)	III	Randomised 1:1, placebo-controlled, added to radiotherapy plus temozolomide	Single blind	716 randomised	60	314 (43.9)
Muragaki, 2011 [11]	Japan	MC (2)	I/II	Single-arm; historical radiotherapy-alone cohort for context	Open label	22 analyzed (24 enrolled)	58	7 (31.8)
Omuro, 2022 [36]	USA	MC (≥8)	I	Non-randomised (part A) and randomised 1:1 (part B) cohorts; no standard-of-care-only arm	NR	136 enrolled / 117 treated	55.0–62.5 by cohort	35 (29.9)
Omuro, 2023 [19]	Multinational (19 countries)	MC (124)	III	Randomised 1:1 versus temozolomide plus radiotherapy	Open label	560 randomised	59.5 versus 56.0	195 (34.8)
Ozer, 2026 [27]	USA	MC (~13)	II	Randomised, placebo-controlled, plus non-randomised ancillary group	Double blind	32 treated (17 randomised)	58.4–64.0*	9 (28.1)
Parney, 2022 [58]	USA	SC (1)	I	Single-arm; no comparator	Open label	21 enrolled / 20 treated	60.5 (28–77)*	9 (42.9)
Phuphanich, 2013 [8]	USA	SC (1)	I	Single-arm; no comparator	Open label	21 enrolled / 20 treated	52 (26–79)	5 (23.8)
Rampling, 2016 [47]	United Kingdom	MC (≥7)	I	Single-arm, two sequential cohorts	Open label	45 treated	53 (20–75)	15 (33.3)
Sampson, 2010 [44]	USA	MC (2)	II	Single-arm; matched contemporaneous cohort (n = 17)	Open label	18 treated	52	5 (27.8)
Schuster, 2015 [45]	USA	MC (24)	II	Single-arm after protocol redesign	Open label	65 treated	56	32 (49.2)
Sim, 2023 [26]	Australia, USA	MC (16)	II	Randomised 2:1 versus temozolomide alone	Open label	103 randomised	73	37 (35.9)
Sloan, 2024 [35]	USA	MC (9)	I	Single-arm, sequential non-randomised dose cohorts	Open label	31 treated	55	10 (32.3)
Wainwright, 2025‡ [17]	USA	MC (≥2)	I	Single-arm, two MGMT-defined cohorts	Open label	18 treated	58 and 64.5 by cohort	7 (38.9)
Wainwright, 2025‡ [17]	USA	MC (≥2)	I	Single-arm, two MGMT-defined cohorts	NR	18 treated	58 and 64.5 by cohort	7 (38.9)
Wang, 2022 [15]	China	SC (1)	I	Single-arm, correlative analysis; no comparator	Open label	19 analyzed (20 vaccinated)	NR	NR
Weathers, 2025 [31]	USA	SC (1)	I/II	Single-arm; external TCGA and GLASS cohorts for validation only	Open label	60 treated	57.8 ± 12.7*	19 (31.7)
Weller, 2017 [21]	Multinational (22 countries)	MC (165)	III	Randomised 1:1 versus keyhole limpet hemocyanin control injection	Double blind	745 randomised	59 versus 57 (MRD)	151 (37.3)†
Wen, 2019 [28]	USA	MC (~19)	II	Randomised 2:1 versus unpulsed autologous dendritic cells	Double blind	124 randomised	59.2 (22.9–81.8)	49 (39.5)

Study (first author, year)	Country	SC/MC (n centres)	Phase	Trial design and comparator	Blinding	Sample size	Median age, years	Female, n (%)
Werlenius, 2021 [24]	Sweden	MC (4)	II	Randomised 2:1 versus standard of care alone	Open label	62 randomised / 61 analyzed	57 (38–69)	25 (41.0)
Wheeler, 2016 [9]	USA	MC (4 + 1 control site)	1b/II	Single-arm; concurrent matched control cohort (n = 134)	Open label	48 treated (+134 controls)	57 (32–72)	NR
Withers, 2025 [32]	USA	MC (5)	II	Single-arm (molecular subset of a 63-patient trial); external TCGA comparison	NR	34 analyzed (63 enrolled)	60 (24–76)	11 (32.4)
Zhang, 2020 [30]	China	SC (1)	I	Single-arm, correlative analysis; internal long- versus short-term survivor comparison	Open label	16 analyzed (20 vaccinated)	52–53 by subgroup	7 (43.8)

Abbreviations: GBM, glioblastoma; ITT, intention-to-treat; IQR, interquartile range; KPS, Karnofsky Performance Status; MC, multicenter; mITT, modified intention-to-treat; MRD, minimal residual disease; NR, not reported; NRG, NRG Oncology National Clinical Trials Network; SC, single center; SOC, standard of care. Values are median (range) unless otherwise indicated. *Reported as mean (± standard deviation or range). †Reported for the whole analyzed population rather than for the subgroup shown in the sample-size column. ‡Two separate records extracted from the same trial (NCT04047706).

Table S2. Survival outcomes, safety and synthesis of findings.

Study (first author, year)	Intervention agent	Median OS, months	Median PFS, months	Grade ≥3 adverse events	Biomarker-based patient selection and relation to toxicity	Synthesis
Ardon, 2010 [53]	Autologous tumour-lysate DC vaccine	24.0 (13–44)	18.0 (2–34)	Three events among 8 patients (12.5% each: haematotoxicity, ischaemic event, status epilepticus); no treatment-related deaths	None; toxicity not analyzed by biomarker	Feasible and well tolerated; immunological responses in most patients but no correlation with clinical outcome; these pilot data powered the subsequent phase I/II trial
Ardon, 2012 [55]	Autologous tumour-lysate DC vaccine	18.3 (ITT); 19.4 (as-treated)	10.4 (ITT); 11.0 (as-treated)	Mainly grade 3 neurological and hematological events, each ≤6.5%; 1 treatment-related death (1.3%)	None; toxicity not analyzed by biomarker	Full integration of DC vaccination into postoperative chemoradiotherapy is safe and possibly beneficial, with the largest signal in patients with minimal residual disease
Bagley, 2023 [16]	CAR T-EGFRvIII plus pembrolizumab	11.8 (90% CI 9.2–14.2)	5.2 (90% CI 2.9–6.0)	Five grade ≥3 events among 7 patients (14.3% each); no treatment-related deaths	MGMT-unmethylated required; EGFRvIII-directed product; toxicity not analyzed by biomarker	Safe and biologically active, with CAR T-cell trafficking to tumour, but without an efficacy signal; alternative strategies are required
Batich, 2020 [12]	CMV pp65 RNA-pulsed DC vaccine	37.7 (ATTAC-GM) and 38.3 (ATTAC-Td) versus 14.9 in historical controls	Not reported	Not reported in this report	None; CMV pp65-directed; safety not reported here	Reproducible long-term survival across three sequential CMV-DC trials, exceeding matched historical and published standard-of-care benchmarks; hypothesis-generating given the non-randomised comparison
Bota, 2022 [54]	AV-GBM-1 DC vaccine	16.0 from enrolment (14.0 from first injection)	10.4 from enrolment (8.5 from first injection)	Frequent CNS events: seizure 12.2%, grade 3 cerebral oedema 10.5% and grade 4 3.6%, neutropenia 7%; no treatment-related deaths	None, but successful tumour-initiating-cell culture required; toxicity not analyzed by biomarker	Reliably manufactured and tolerable but with numerous treatment-emergent CNS events; PFS exceeded historical benchmarks without a gain in OS
Buchroithner, 2018 [25]	Audencel DC vaccine	18.8 versus 18.9 in controls	6.8 versus 7.0 in controls	Grade 3–4 thrombocytopenia 20.5% versus 5% (the only significant between-arm difference); no vaccine-attributable deaths	None, but sufficient tumour protein required; the only between-arm difference, grade 3–4 thrombocytopenia, was unrelated to biomarker status	Negative randomised trial: no PFS or OS benefit despite feasibility, good tolerability and induction of tumour-directed immune responses
Bunse, 2026 [46]	IDH1-vac peptide vaccine	Not reached at 8 years (8-year OS 66%); 106.1 in grade IV tumors	60.1 (95% CI 32.3–NE)	Not restated (reported in the companion primary publication); no adverse events with booster dosing	IDH1-R132H-positive, 1p/19q non-codeleted, ATRX-lost required; safety reported in the primary publication	Long-term follow-up shows durable peripheral and intratumoral immune responses and favorable outcome, particularly with complete remission; not designed to demonstrate efficacy
Chen, 2025 [43]	Tumour Treating Fields plus pembrolizumab plus temozolomide	24.8 (per protocol) versus 14.6 in matched controls	12.0 versus 5.8 in matched controls	16 of 213 treatment-related events (7.5%) were grade ≥3; treatment-related deaths not reported	None; MGMT status recorded but not an eligibility criterion; toxicity not analyzed by biomarker	Suggests synergy between Tumour Treating Fields and checkpoint blockade, greatest in patients with high tumour burden; based on a non-randomised matched comparison
Cho, 2012 [13]	Autologous tumour-lysate DC vaccine	31.9 versus 15.0 in controls (p<0.002)	8.5 versus 8.0 in controls (p=0.075)	No formal CTCAE grading; isolated surgical and hematological events; no treatment-related deaths	None; toxicity not analyzed by biomarker	Randomised evidence of improved short-term survival with DC vaccination; small, single-center and unblindable design limits inference
Fadul, 2024 [60]	EGFR BATs	28.8	17.2	No dose-limiting toxicities across 68 infusions; grade 3–4 lymphopenia in 21 of 68 infusions (31%); no treatment-related deaths	None, but molecular MGMT confirmation and absence of elevated alpha-gal IgE required; toxicity not analyzed by biomarker	The maximum feasible dose was safe and induced tumour-specific immune responses, supporting efficacy testing in MGMT-unmethylated disease

Study (first author, year)	Intervention agent	Median OS, months	Median PFS, months	Grade ≥3 adverse events	Biomarker-based patient selection and relation to toxicity	Synthesis
Fares, 2021 [10]	NSC-CRAd-S-pk7 oncolytic virus	18.4	9.1	Grade 3 lymphopenia and hypertension 42% each, muscle weakness 33%; two grade 4 events in one patient; no treatment-related deaths	None; toxicity not analyzed by biomarker	Feasible and safe intra-cavitary delivery with supportive immunological and histopathological findings; phase 2/3 evaluation is warranted
Gentner, 2026 [29]	Temferon (TIE2–interferon-α CD34⁺ gene therapy)	16.7 from diagnosis	8.1 from diagnosis	17.4% of events grade 3 and 7.0% grade 4; grade 5 events in 4 of 24 patients (16.7%); 3 of 24 (12.5%) died of conditioning-related infection	MGMT-unmethylated required; the highest toxicity in this review, driven by myeloablative conditioning rather than by biomarker status	Safe and feasible up to 4×10^6 cells/kg as first-line therapy with dose-dependent transgene marking; toxicity was driven mainly by the conditioning regimen
Han, 2022 [23]	Autologous CIK cells	23.1 versus 14.9 in controls (pure GBM)	8.1 versus 5.5 in controls	Grade ≥3 AEs 47.1% versus 36.5% (not significant); no treatment-related deaths	Post hoc restriction to pathologically pure GBM; grade ≥3 AEs 47.1% versus 36.5%, not significant	In pathologically pure GBM, CIK immunotherapy prolonged OS and PFS on multivariable analysis, although the Kaplan–Meier OS difference was not significant
Hilf, 2019 [48]	APVAC1 and APVAC2 personalized vaccines	29.0 from diagnosis	14.2 from diagnosis	Grade ≥3 AEs in 11 of 16 patients (68.8%), mainly temozolomide-related marrow suppression; no treatment-related deaths	HLA-A*02:01 and/or A*24:02 required; grade ≥3 events mainly temozolomide-related and not HLA-dependent	Feasible individualized vaccination with favorable safety and strong immunogenicity: sustained CD8⁺ responses to unmutated antigens and CD4⁺ Th1 responses to mutated antigens
Inogés, 2017 [56]	Tumour-lysate-pulsed DC vaccine	23.4	12.7	Severe events (7.9% of all AEs) attributed to standard therapy; no vaccine-related deaths	None, but complete 5-ALA-guided resection required; severe events attributed to standard therapy	Feasible and safe addition to maximal resection and chemoradiotherapy; the potential survival benefit requires randomised confirmation
Ishikawa, 2014 [63]	Autologous formalin-fixed tumour vaccine	22.2	8.2	Grade 3–4 events predominantly chemoradiotherapy-attributed lymphopenia and neutropenia; not aggregated per patient	None; toxicity chemoradiotherapy-attributed	Integration with the Stupp regimen was tolerable with encouraging survival, supporting controlled evaluation
Jacques, 2021 [34]	Avelumab	15.3	9.7	Immune-related AEs in 8 of 30 patients (26.7%), of which 22.6% were grade ≥3; infusion-related reactions in 26.6%; no treatment-related deaths	None; unselected for PD-L1 expression; immune-related AEs in 26.7%, not biomarker-stratified	No new safety signal but no apparent improvement in OS over standard therapy
Ji, 2018 [14]	HSPPC-96	31.4	11.0	One of 20 patients (5%) with a grade 3 event, judged unrelated to the vaccine; no treatment-related deaths	None; toxicity not analyzed by biomarker	Safe and immunogenic; the post-vaccination tumour-specific immune response predicted vaccine efficacy
Johanns, 2024 [50]	NeoVax with poly-ICLC	Not reported (individual OS 195–497 days)	Not reported (individual PFS 9–15 months)	No grade ≥3 events; no treatment-related deaths	MGMT-unmethylated on a CLIA-certified assay required, with patient-specific neoantigens needed for manufacture; no grade ≥3 events	Safety endpoint met but the 4-week feasibility endpoint was not (mean production time 164 days); multisector sampling expanded the neoantigen pool by approximately 40%

Study (first author, year)	Intervention agent	Median OS, months	Median PFS, months	Grade ≥3 adverse events	Biomarker-based patient selection and relation to toxicity	Synthesis
Kesari, 2024 [42]	Nivolumab plus ipilimumab before radiotherapy	19.3	1.3	Grade 3 rash 20% and lipase elevation 13%; one grade 4 cerebral oedema (7%); no grade 5 events	None; toxicity not analyzed by biomarker	Pre-radiotherapy checkpoint blockade is safe and operationally feasible; the very short PFS reflects the untreated window design
Keskin, 2019 [49]	Personalised neoantigen vaccine	16.8	7.6	No grade ≥3 events; no treatment-related deaths	MGMT-unmethylated required, with at least 5 actionable neoepitopes; no grade ≥3 events	Feasible in a tumour with low mutational burden; vaccination generated circulating and intratumoral neoantigen-specific T cells, yet all patients progressed
Kong, 2017 [22]	Autologous CIK cells	22.5 versus 16.9 in controls (not significant)	8.1 versus 5.4 in controls	Grade 3 adverse drug reactions ≤1.2% each; no grade 4 events; no treatment-related deaths	None; no grade 4 events; toxicity not analyzed by biomarker	Randomised phase III: PFS improved with CIK cells but without an OS benefit, and no subgroup with selective benefit was identified
Lassman, 2025 [18]	Ipilimumab plus nivolumab without temozolomide	Approximately 13 in both arms (data immature)	7.7 versus 8.5 with temozolomide	Grade 3–5 AEs 41.0% versus 37.8% (not significant); two treatment-related deaths (2.6%) on the immunotherapy arm	MGMT-unmethylated and IDH wild-type required; grade 3–5 AEs 41.0% versus 37.8%, not significant	Negative randomised trial: dual checkpoint blockade did not improve PFS in MGMT-unmethylated GBM; accrual closed and the trial will not proceed to phase III
Lim, 2022 [20]	Nivolumab with radiotherapy plus temozolomide	28.9 versus 32.1 with placebo	10.6 versus 10.3 with placebo (blinded central review)	Grade 3–4 treatment-related AEs 52.4% versus 33.6%; four treatment-related deaths (1.1%) versus none	MGMT-methylated or indeterminate promoter required; grade 3–4 treatment-related AEs 52.4% versus 33.6%, driven by checkpoint blockade rather than by methylation status	Largest phase III in MGMT-methylated or indeterminate GBM: nivolumab did not improve PFS or OS and increased toxicity
Muragaki, 2011 [11]	Autologous formalin-fixed tumour vaccine	21.4	7.6	No events exceeding grade 1 and no hematological toxicity; no treatment-related deaths	None; no events exceeding grade 1	Vaccination concomitant with radiotherapy may be effective without severe toxicity, supporting evaluation in combination with temozolomide
Omuro, 2022 [36]	Nivolumab with radiotherapy, with or without temozolomide	33.4 (MGMT-methylated/unknown) and 16.5 (unmethylated) with temozolomide; 14.4 without temozolomide	15.5 and 6.5 with temozolomide; 5.6 without temozolomide	Grade 3–4 treatment-related AEs 51.6% (with temozolomide) versus 30.0% (radiotherapy alone) in part A; one grade 5 event	None; MGMT status recorded but unselected; grade 3–4 AEs 51.6% with temozolomide versus 30.0% without	First trial of checkpoint blockade in first-line GBM: nivolumab can be safely combined with radiotherapy with or without temozolomide, with outcomes differing by MGMT status
Omuro, 2023 [19]	Nivolumab plus radiotherapy versus temozolomide plus radiotherapy	13.4 versus 14.9 with temozolomide	6.0 versus 6.2 with temozolomide	Grade 3–4 treatment-related AEs 21.9% versus 25.1%; three treatment-related deaths (1.1%) versus none	MGMT-unmethylated and IDH wild-type required; grade 3–4 treatment-related AEs 21.9% versus 25.1%	Negative phase III: nivolumab did not improve OS over temozolomide in MGMT-unmethylated GBM, confirming temozolomide plus radiotherapy as standard of care
Ozer, 2026 [27]	Pembrolizumab plus temozolomide plus HSPPC-96	14.4 (vaccine) versus 15.2 (placebo) versus 8.8 (ancillary)	9.9 versus 7.9 versus 7.4	Grade 3–4 AEs in 21 of 32 patients (66%), treatment-attributable in 50–60% per group	IDH wild-type and MGMT-unmethylated at registration; grade 3–4 AEs 66%, similar across randomised groups	Trial discontinued early; findings do not support adding pembrolizumab plus HSPPC-96 to standard therapy in MGMT-unmethylated GBM
Parney, 2022 [58]	Allogeneic-lysate-pulsed autologous DC vaccine	19.0	9.7	Grade 3–4 lymphopenia 35%; neutropenia, thrombocytopenia and leukopenia 10% each; no treatment-related deaths	HLA-A0201 required for the first 10 patients; toxicity dominated by lymphopenia and not HLA-dependent	Safe and highly feasible manufacturing platform; imaging, histological and immunological data suggest a positive biological response

Study (first author, year)	Intervention agent	Median OS, months	Median PFS, months	Grade ≥3 adverse events	Biomarker-based patient selection and relation to toxicity	Synthesis
Phuphanich, 2013 [8]	ICT-107 DC vaccine	38.4 (newly diagnosed subgroup)	16.9 (newly diagnosed subgroup)	No grade ≥3 events; only grade 1–2 AEs reported	HLA-A1 or HLA-A2 positivity and expression of at least one vaccine antigen required; no grade ≥3 events	Expression of four targeted antigens correlated with prolonged OS and PFS; loss of CD133 at recurrence supports targeting cancer stem-cell antigens
Rampling, 2016 [47]	IMA950 with GM-CSF	15.3 overall (14.4 and 15.7 by cohort)	Not reported (presented as 6- and 9-month PFS rates)	Two of 45 patients (4.4%) with dose-limiting grade 3 events; grade 3–4 seizures in 5 of 45 (11%); no treatment-related deaths	HLA-A*02 positivity required; dose-limiting grade 3 events in 4.4%; toxicity not HLA-stratified	Well tolerated and exceeded the prespecified multi-peptide immunogenicity threshold, supporting further development of the vaccine
Sampson, 2010 [44]	PEPvIII-KLH	26.0 versus 15.0 in the matched cohort	14.2 versus 6.3 in the matched cohort	Minimal toxicity, mainly injection-site reactions never exceeding grade 1; no treatment-related deaths	EGFRvIII positivity on central immunohistochemistry required; toxicity minimal and confined to injection-site reactions	Encouraging survival with EGFRvIII-targeted vaccination and evidence of antigen-loss escape; warranted a randomised phase III trial
Schuster, 2015 [45]	Rindopepimut with GM-CSF	21.8 from study entry (24.6 from diagnosis)	9.2 from study entry (12.3 from diagnosis)	Grade 3–4 treatment-related events rare (≤2% each); no fatal adverse events	EGFRvIII positivity required; grade 3–4 treatment-related events ≤2% each	Confirmed the reproducibility of earlier phase II results in a multicenter setting, prompting the pivotal ACT IV trial
Sim, 2023 [26]	Nivolumab plus temozolomide in elderly patients	11.6 versus 11.8 with temozolomide	7.4 versus 5.9 with temozolomide	Grade ≥3 AEs 46% versus 29%; no treatment-related deaths	None; age ≥65 years; grade ≥3 AEs 46% versus 29%	Insufficient evidence of benefit in elderly patients receiving hypofractionated chemoradiotherapy; the trial did not progress to phase III
Sloan, 2024 [35]	Ipilimumab and/or nivolumab with temozolomide	20.7 (combination cohort)	16.1 (combination cohort)	Highest-grade related AEs: grade 3 in 16.7–38.5% and grade 4 in 0–16.7% across cohorts; no grade 5 events	None; highest-grade related AEs grade 3 in 16.7–38.5% across cohorts, unrelated to biomarker status	Combination checkpoint blockade with adjuvant temozolomide is safe and no more toxic than single agents, supporting a subsequent efficacy trial
Wainwright, 2025a‡ [17]	Nivolumab plus BMS-986205 (IDO1 inhibitor)	11.5 (cohort A) and 26.9 (cohort B)	7.7 (cohort A) and 13.1 (cohort B)	Cohort A: grade 3–4 AEs 75% and grade 5 events 33.3%; dose-limiting transaminase elevation in 5 of 12; cohort B: cerebral oedema the only dose-limiting toxicity	IDH wild-type required, with cohorts defined by MGMT status; grade 3–4 AEs 75% and grade 5 events 33.3% in the unmethylated cohort A, against cerebral oedema as the only dose-limiting toxicity in the methylated cohort B	Safe and tolerable overall; the results support further study of mechanisms of action, resistance and predictive biomarkers
Wainwright, 2025b‡ [17]	Nivolumab plus BMS-986205 (IDO1 inhibitor)	11.5 (cohort A) and 26.9 (cohort B)	7.7 (cohort A) and 13.1 (cohort B)	Cohort A: any AE 100%, grade 3–4 AEs 75% and grade 5 events 33.3%; cohort B: cerebral oedema the only dose-limiting toxicity	IDH wild-type required, with cohorts defined by MGMT status; any AE 100% and grade 3–4 AEs 75% in the unmethylated cohort A, against cerebral oedema as the only dose-limiting toxicity in the methylated cohort B	Concurrent radiotherapy, nivolumab and IDO1 inhibition were tolerable; mechanisms of action, treatment resistance and predictive biomarkers warrant further investigation
Wang, 2022 [15]	HSPPC-96	31.4	11.0	Not assessed (correlative sub-study)	Restricted to patients vaccinated in the parent trial with pre-vaccination tissue; low MxA expression predicted better outcome; safety not assessed	Low pre-vaccination MxA expression correlated with better survival, identifying a candidate biomarker for pre-vaccination selection of responders
Weathers, 2025 [31]	Atezolizumab with radiotherapy plus temozolomide	18.0 overall; 16.1 in IDH wild-type patients	Not reported	Grade ≥3 treatment-related AEs in 34 of 60 patients (56.7%), most commonly lymphopenia (38.3%)	Explicitly unselected for MGMT methylation; grade ≥3 treatment-related AEs 56.7%, mainly lymphopenia	Safe with modest efficacy; EGFR-mutant tumors fared worse and immune enrichment tracked with the pre-treatment mesenchymal subtype

Study (first author, year)	Intervention agent	Median OS, months	Median PFS, months	Grade ≥3 adverse events	Biomarker-based patient selection and relation to toxicity	Synthesis
Weller, 2017 [21]	Rindopepimut versus keyhole limpet haemocyanin	20.1 versus 20.0 in controls (minimal residual disease)	8.0 versus 7.4 in controls (minimal residual disease)	Grade 3–4 thrombocytopenia 9% versus 6%; one treatment-related death (0.3%) versus none	Centrally confirmed EGFRvIII positivity required; grade 3–4 thrombocytopenia 9% versus 6%	Definitive negative phase III: rindopepimut did not prolong survival despite robust EGFRvIII-specific antibody responses
Wen, 2019 [28]	ICT-107 DC vaccine	17.0 versus 15.0 in controls	11.2 versus 9.0 in controls	No difference in overall AE rates; grade 3 convulsion 5.0% versus 2.3%	HLA-A1 and/or HLA-A2 positivity required; no difference in overall AE rates between arms	PFS was significantly improved with preserved quality of life; the greatest clinical and immunological activity was seen in the HLA-A2 subgroup
Werlenius, 2021 [24]	ALECSAT	19.2 versus 18.3 with standard of care	7.8 versus 7.9 with standard of care	Grade ≥3 AEs in 92.5% versus 81.0% of patients; no treatment-related deaths in the ALECSAT arm	None; grade ≥3 AEs 92.5% versus 81.0%	Well tolerated but without a PFS or OS benefit; concomitant temozolomide and corticosteroids may have blunted efficacy
Wheeler, 2016 [9]	AdV-tk with valacyclovir	17.1 versus 13.5 in matched controls (25.1 versus 16.3 after gross total resection in GBM)	8.1 versus 6.5 in matched controls	No dose-limiting toxicities; grade >2 neurological events in 16% during weeks 0–3; treatment-related deaths not reported	None; no dose-limiting toxicities	Survival improved versus a prespecified matched control cohort, particularly after maximal debulking; randomised confirmation is required
Withers, 2025 [32]	SurVaxM with temozolomide	25.9 in the whole trial (25.6 in the profiled subset)	11.4 in the whole trial	Not assessed (correlative study)	Restricted to SurVaxM-treated patients with pre-treatment tissue; B-cell and interferon signatures predicted survival; safety not assessed	Sensitivity to vaccination tracked with pre-existing tumour immune status, specifically interferon-responsive gene signatures and B-cell infiltration
Zhang, 2020 [30]	HSPPC-96 with temozolomide	Not reached with high post-vaccination TSIR versus 14.6 with low TSIR	12.3 versus 9.0 by TSIR group	Not assessed (correlative study)	Restricted to vaccinated patients; T-cell receptor repertoire predicted survival; safety not assessed	The intratumoral T-cell receptor repertoire differed markedly between long- and short-term survivors; shared expanded clones are proposed as response biomarkers

Abbreviations: AE, adverse event; CI, confidence interval; CMV, cytomegalovirus; CNS, central nervous system; CTCAE, Common Terminology Criteria for Adverse Events; DC, dendritic cell; GBM, glioblastoma; GM-CSF, granulocyte–macrophage colony-stimulating factor; ITT, intention-to-treat; NE, not estimable; OS, overall survival; PFS, progression-free survival; TSIR, tumour-specific immune response. ‡Two separate records extracted from the same trial (NCT04047706). EGFRvIII, epidermal growth factor receptor variant III; HLA, human leukocyte antigen; IDH, isocitrate dehydrogenase; IDO1, indoleamine 2,3-dioxygenase 1; MGMT, O^6-methylguanine-DNA methyltransferase; 5-ALA, 5-aminolevulinic acid.
