# Supplementary material for: Immunotherapy for Newly Diagnosed Glioblastoma: Current Evidence and Future Perspectives
Source: J Pers Med. 2026 Sep 17;16(9):479. doi: 10.3390/jpm16090479 (PMC13608823; doi:10.3390/jpm16090479)
Supplement: Supplementary file 1 [file jpm-16-00479-s001.zip › jpm-4523339-supplementary-PRISMA-ScR Checklist.pdf]

PRISMA-ScR Checklist

Manuscript: "Advancing the Management of Glioblastomas: the Potential of Immunotherapy and Future Perspectives" (scoping review). Page numbers refer to the submitted manuscript.

#	PRISMA-ScR item	Where it is reported	Page
1	Title	Identifies the report as a scoping review	**p. 1**
2	Abstract	Structured summary (background, methods, results, conclusion)	**p. 1**
3	Rationale	Rationale for the review in the context of what is known	**p. 2**
4	Objectives	Review question and objectives (PCC-based)	**pp. 2-3**
5	Protocol and registration	Registered on Figshare (10.6084/m9.figshare.33189945); no major deviations	**p. 2**
6	Eligibility criteria	PCC framework; inclusion and exclusion criteria	**p. 3**
7	Information sources	PubMed, Scopus, Embase, Web of Science; inception to July 2026	**p. 3**
8	Search	Full electronic search strategy reported	**p. 3**
9	Selection of sources of evidence	Two-stage screening, two independent reviewers, Rayyan, third reviewer for disagreements	**p. 3**
10	Data charting process	Charting by two authors using predefined criteria	**p. 3**
11	Data items	Study, population, tumour, therapy and outcome variables listed	**p. 3**
12	Critical appraisal of individual sources	Not performed (optional in PRISMA-ScR); explicitly stated	**p. 9**
13	Synthesis of results	Tabular and diagrammatic mapping with structured narrative synthesis	**pp. 3-4**
14	Selection of sources of evidence	21,304 screened, 91 full texts, 45 included; PRISMA flow diagram (Figure 1)	**p. 4**
15	Characteristics of sources of evidence	Design, phase, centres, sample, age, sex (Table 1); eligibility and intervention (Table 2)	**pp. 4-5; Tables 1-2, pp. 13-21**
16	Critical appraisal within sources	Not applicable – no formal risk-of-bias assessment performed	**p. 9**
17	Results of individual sources of evidence	OS, PFS, grade ≥3 adverse events and synthesis per study	**pp. 5-6; Table 3, pp. 22-26**
18	Synthesis of results	Results grouped by therapeutic class and by outcome domain	**pp. 5–6**
19	Summary of evidence	Main findings by modality, linked to review questions	**pp. 6–9**
20	Limitations	Early-phase designs, heterogeneity, no risk-of-bias assessment, publication bias	**p. 9**
21	Conclusions	Overall interpretation and implications for future trials	**p. 9**
22	Funding	No external funding; no conflicts of interest declared	**p. 9**
